# ROS-responsive MnO_2_ mesoporous hydrogel to modulate liver-muscle crosstalk and mitigate NAFLD-associated sarcopenia via exosomal miR-582-5p delivery

**DOI:** 10.7150/thno.108280

**Published:** 2025-03-21

**Authors:** Songling Jiang, Tae Min Kim, Sung Young Park, Eun-Jung Jin

**Affiliations:** 1 Department of Biomedical Materials Science, Graduate school of JABA, Wonkwang University, Iksan, Jeonbuk State, 54538, Republic of Korea.; 2 Department of IT and Energy Convergence (BK21 FOUR), Korea National University of Transportation, Chungju 27469, Republic of Korea.; 3 Department of Chemical and Biological Engineering, Korea National University of Transportation, Chungju 27469, Republic of Korea.; 4 Integrated Omics Institute, Wonkwang University, Iksan, Jeonbuk State, 54538, Republic of Korea.

**Keywords:** Hydrogel, NAFLD, Sarcopenia, ROS, miR-582-5p, Exosomal Delivery

## Abstract

**Background:** The interrelation between non-alcoholic fatty liver disease (NAFLD) and sarcopenia has emerged as a significant concern due to its systemic impact on metabolic health. However, therapeutic approaches targeting the liver-muscle axis remain underdeveloped. Oxidative stress and inflammatory pathways are key mediators of this crosstalk, exacerbating disease progression. This study aims to develop a reactive oxygen species (ROS)-responsive MnO_2_ mesoporous PD (HA) hydrogel to modulate this axis and investigate its therapeutic efficacy in NAFLD-associated sarcopenia.

**Methods:** MnO_2_ mesoporous PD (HA) hydrogels were synthesized with ROS-sensitive properties and characterized for rheological, fluorescence, and conductivity responses. A HepG2-C2C12 co-culture model mimicked the NAFLD-muscle wasting interplay, while high-fat diet (HFD)-induced NAFLD mouse models were used for in vivo evaluations. Cellular stress markers, exosomal miR-582-5p signaling, and atrogenic pathways were assessed using immunofluorescence, qRT-PCR, and histological analyses.

**Results**: Pathway analysis of HFD-induced NAFLD showed upregulation of lipid metabolism and inflammatory signaling, promoting muscle atrophy via exosomal miR-582-5p. The MnO_2_ mesoporous hydrogel significantly reduced oxidative stress and inflammation in the HepG2-C2C12 co-culture. In vivo, hydrogel implantation in HFD mice mitigated hepatic fibrosis, reduced ROS accumulation, preserved muscle fiber integrity, and downregulated atrogenic markers.

**Conclusions:** The MnO_2_ mesoporous PD (HA) hydrogel presents a dual-targeting therapeutic strategy for NAFLD and sarcopenia by attenuating oxidative stress and modulating liver-muscle axis signaling. These findings provide a foundation for innovative interventions targeting metabolic comorbidities.

## Introduction

Non-alcoholic fatty liver disease (NAFLD) encompasses a wide range of liver pathologies, from simple hepatic steatosis to more severe non-alcoholic steatohepatitis (NASH), fibrosis, and eventually cirrhosis 1. NAFLD has emerged as a significant global health issue, primarily because of its rising prevalence in parallel with increasing rates of obesity, type 2 diabetes mellitus (T2DM), and other components of metabolic syndrome, particularly in developed nations 2. NAFLD affects approximately 25 % of the global population, with even higher prevalence rates reported in specific demographic groups such as those with T2DM 3. The progression of NAFLD is closely associated with metabolic dysregulation, which exacerbates liver damage and predisposes individuals to cardiovascular diseases, further compounding the health burden 4. Concurrently, sarcopenia, defined as the progressive loss of skeletal muscle mass and strength, has gained recognition as another critical public health concern 5. The prevalence of sarcopenia increases with age, leading to a significant decline in physical function and quality of life and is often associated with an increased risk of falls, frailty, and mortality 6. Recent evidence suggests a bidirectional relationship between NAFLD and sarcopenia, wherein they exacerbate each other, forming a detrimental cycle that worsens the overall health outcomes 7,8. This relationship is believed to be mediated through the liver-muscle axis, indicating a complex interplay of metabolic signals between these two tissues. Liver dysfunction, as observed in NAFLD, can disrupt muscle metabolism by altering nutrient availability, chronic inflammation, and increasing oxidative stress, thereby contributing to the development and progression of sarcopenia.

Oxidative stress is the central feature in the pathogenesis of both NAFLD and sarcopenia and is characterized by an imbalance between the production of reactive oxygen species (ROS) and antioxidant defenses in the body 9,10. Excessive ROS production in the liver leads to hepatocyte injury, inflammation, and subsequent fibrogenesis, driving progression from simple steatosis to NASH and fibrosis 11. In skeletal muscles, elevated ROS levels impair mitochondrial function and protein synthesis, leading to muscle atrophy and impaired regenerative capacity, which are hallmarks of sarcopenia 12. Given the role of oxidative stress in both conditions, therapeutic strategies that can mitigate ROS accumulation hold promise for addressing the intertwined pathologies of NAFLD and sarcopenia. Recent advances in nanotechnology have highlighted the potential of manganese dioxide (MnO_2_) mesoporous nanoparticles as potent ROS scavengers owing to their unique structural and catalytic properties. These nanoparticles have been shown to efficiently neutralize ROS, thereby protecting cells from oxidative damage 13. In this study, we investigated the therapeutic potential of MnO_2_ mesoporous PD (M-PD) hydrogel (Exp-gel) in addressing both NAFLD and sarcopenia through the liver-muscle axis. We hypothesized that administration of mesoporous Exp-gel would lead to a significant reduction in hepatic oxidative stress, thereby attenuating the progression of NAFLD. This reduction in oxidative stress is expected to promote the release of exosomes enriched with miR-582-5p, a miRNA known for its anti-atrogenic properties. When delivered to the skeletal muscle, these exosomes are anticipated to inhibit the expression of atrogenic genes that are involved in muscle protein degradation, thereby improving muscle mass and function. This dual therapeutic strategy, targeting NAFLD and sarcopenia simultaneously, represents a novel approach for treating these interconnected conditions. By focusing on the liver-muscle axis and utilizing the advanced properties of mesoporous Exp-gel, our study aims to contribute to a growing body of evidence supporting the use of targeted nanotherapeutics in complex metabolic diseases. The findings of this study could pave the way for new interventions that not only address the underlying oxidative stress but also offer a comprehensive solution to the multifactorial challenges posed by NAFLD and sarcopenia.

## Methods

### Characterization of samples

The rheological characteristics of the hydrogels were assessed using a rheometer (Haake Maars, Thermo Fisher, Germany). An ECLIPSE Ti2-E confocal microscope (Nikon) was used to acquire the CLSM images. The electrochemical properties of the samples were investigated using EIS (CS350, CorrTest Instruments, China) and a source meter (Keithley 2450, Tektronik, USA). A smartphone was used to present real-time data and an Arduino Uno microcontroller (ATmega328 P Processor) was employed for wireless monitoring. The Arduino Uno and smartphone could connect wirelessly owing to the AppGosu Bluetooth module.

### Synthesis of MnO_2_ meso@PD (HA) and MnO_2_ nanosheet-PD (HA)

Hyaluronic acid (HA; Mw = 100,000 Da), gallic acid monohydrate, polyvinyl alcohol PVA (Mw = 125,000 Da), and potassium permanganate (KMnO_4_) were purchased from Sigma-Aldrich (Korea). Phosphate-buffered saline (PBS, pH 7.4) was purchased from Bioneer Corp. The synthesis of dopamine-conjugated hyaluronic acid polymer dots [PD (HA)], MnO_2_ mesoporous nanoparticles, and gallic acid-conjugated chitosan (CHI-GA) has been described in a previous report 14-16. To prepare the MnO_2_ meso@PD (HA) nanoparticles, 30 mg of PD (HA) was mixed with 10 mL of TBS (pH 8.5) and stirred at room temperature. After dissolving until a homogeneous state was reached, 3 mg of MnO_2_ meso was added to the mixture and stirred for 24 h. The mixture was dialyzed (3.5 kD) against double deionized water (DDW), and the final solution was lyophilized to obtain MnO_2_ meso@PD (HA). PD (HA) (30 mg) and KMnO_4_ solution (5 mM, 3.8 mL) were mixed with MES 0.1 mM (26.2 mL). The mixture was then sonicated for 2 h at room temperature. The sample was then centrifuged and freeze-dried to obtain MnO_2_ nanosheet-PD (HA) [MnO_2_ sheet-PD (HA)].

### Preparation of ROS-responsive hydrogel

Chitosan (low molecular weight), gallic acid monohydrate, and polyvinyl alcohol (PVA, Mw = 125,000 Da) were purchased from Sigma-Aldrich (Korea). 0.5 g of PVA was mixed with DDW (3.9 mL) and stirred at 90 ℃ until a homogeneous state was achieved. The MnO_2_ meso@PD (HA) solution or MnO_2_ sheet-PD (HA) solution (50 mg in 1.1 mL of DDW) was mixed with the above mixture and stirred at room temperature until a homogeneous state was achieved. CHI-GA (50 mg) was used to enhance the adhesive properties, and the mixture was stirred for approximately 2 h. The final mixture was poured into a mold and subjected to freeze (18 h)-thaw (6 h) for three cycles. The resulting MnO_2_ meso@PD (HA) hydrogels (Exp-gel) were stored in a refrigerator until further use. In addition, PVA (0.5 g) was melted homogeneously with DDW (3.9 mL) at 90 ℃ and CHI-GA (50 mg) was added gradually at room temperature and stirred for approximately 2 h. The resulting hydrogel was then subjected to freezing-thawing for 3x cycles to obtain a control hydrogel (Con-gel) without ROS-responsive properties.

### Conductivity response of ROS-responsive hydrogel

The conductivity changes of the hydrogel before and after treatment were measured using EIS with a two-electrode system in the frequency range of 10^-1^-10^4^ Hz and a measurement potential of 0 V vs open circuit potential (OCP). The source meter resistances of the hydrogels were measured using a 2-electrode DC system.

### Antioxidant property of hydrogel

In order to confirm the antioxidant property of the hydrogel, both the DPPH assay and the •OH scavenging assay were performed. For the DPPH assay, 3 mL of DPPH solution (0.1 mM) and hydrogel were incubated at 37 °C for 45 minutes. The hydroxyl radical scavenging assay involved the mixture of hydrogel with 1 mL of salicylic acid (6 mM), phosphate-buffered saline (PBS, pH 7.4), FeSO_4_ (6 mM, 1 mL), and H_2_O_2_ (0.01%, 0.5 mL), followed by incubation at 37 °C for 30 minutes. The scavenging activity was then calculated by measuring the optical density of the incubated assay solution using UV-Vis spectroscopy at the wavelengths of 517 nm (DPPH assay) and 505 nm (•OH scavenging assay).

### C2C12 and HepG2 cell culture

C2C12 and HepG2 cells were purchased from American Type Culture Collection (Manassas, VA, USA). C2C12 cells were cultured in high-glucose Dulbecco's modified Eagle's medium (DMEM; Gibco, Grand Island, NY, USA) supplemented with 10% fetal bovine serum (Gibco). In contrast, HepG2 cells were cultured in low-glucose DMEM supplemented with 10% bovine calf serum (Gibco). All cell lines were incubated at 37 °C in a humidified incubator with an atmosphere of 5 % CO_2_. During the differentiation process, C2C12 cells were maintained in high-glucose DMEM supplemented with 2 % horse serum for five days. Once differentiation was complete, the C2C12 myotubes were used for subsequent experiments. For metabolic stress induction, HepG2 cells were treated with either 250 µM palmitic acid (PA) for 6 hours or 200 µM hydrogen peroxide (H₂O₂) for 4 hours. Following treatment, HepG2 cells were cultured with either control gel (Con-gel) or experimental gel (Exp-gel) and subsequently co-cultured with differentiated C2C12 myotubes in 2 mL of fresh medium for 24 hours. For the conditioned medium study, lipid-stressed HepG2 cells were washed and incubated in fresh medium for 24 hours. The resulting conditioned medium was collected, centrifuged at 1300 rpm for 5 minutes, and then used to culture differentiated C2C12 myotubes in 1 mL of conditioned medium for 24 hours.

### BODIPY 493/503 staining

HepG2 cells were washed with PBS and subsequently fixed in 4 % paraformaldehyde (PFA). For BODIPY 493/503 staining, fixed cells were washed thrice with PBS and incubated in PBS containing BODIPY 493/503 dye (1:500, Thermo Fisher Scientific Korea Ltd., D3922) for 30 min. The nuclei were stained using a mounting medium that contained DAPI (Vector Laboratories).

### Immunofluorescence (IF) staining

The cells cultured on coverslips were fixed with 4 % PFA for 10 min. Following fixation, the cells were washed thrice with PBS. Subsequently, the cells were blocked with 3% normal goat serum (NGS; Vector Laboratories, Newark, CA, USA) and incubated with an anti-myosin skeletal heavy chain antibody (MYHC, 1:100; R&D Systems, Minneapolis, MN, USA, #MAB4470) and myosin heavy chain type I antibody (1:100; Developmental Studies Hybridoma Bank, #BA-D5), and with a fluorescence-conjugated secondary antibody. Nuclei were stained using a DAPI-containing mounting medium.

### Animal studies

The animal protocol for this study was approved by the Institutional Animal Care and Use Committee at Wonkwang University and adhered to the institutional guidelines (WKU23-13). The mice were maintained at 23 ± 1 °C, under a 12-h light/dark cycle, and at a relative humidity of 50 ± 5 % food and water were provided ad libitum. Mice were administered either a low-fat diet or high-fat diet (HFD). After 12 weeks on the HFD, Con-gel and Exp-gel were introduced into the liver tissue for 4 weeks.

### ELISA analysis

Mouse blood was collected using a heparin-coated syringe and centrifuged at 4000 rpm for 20 minutes to obtain plasma. Plasma levels of insulin and alanine aminotransferase (ALT) were measured using ELISA kits (80-INSMS-E01 for insulin and MBS264717 for ALT). Liver and quadriceps muscle tissues were homogenized in a 5 % NP-40 buffer at a concentration of 100 mg/µL, and triglyceride levels were quantified using the EZ-Triglyceride Quantification Assay kit (DG-TGC100).

### Histological & oil-red O staining

For histological analysis, the excised muscle tissues were fixed in 10% neutral-buffered formalin prior to being embedded in paraffin or OCT compounds. For hematoxylin and eosin (H&E) staining, 5 µm sections of paraffin-embedded muscle were utilized. Frozen liver, Quadriceps muscle (Qua. muscle), and tibialis anterior (TA) muscle sections were stained with oil-red O (Sigma-Aldrich) in 60 % isopropanol. Staining intensity was quantified using Image-Pro Plus software.

### Exosome isolation

After a 16-week HFD intervention, both normal and fatty liver tissues were isolated and subsequently incubated in RPMI 1640 medium (Gibco) containing 2 mg/mL type 2 collagenase (Worthington Biochemical Corp., NJ, USA) and 40 U/mL DNase I (Roche, Basel, Switzerland) at 37 °C for 30 min. Following differential centrifugation, the supernatants were filtered through a 0.45-μm membrane and then subjected to centrifugation at 100,000 g for 70 min.

### Preparation of *Atrogin-1*- and *MuRF-1*-conjugated PD-Cu^2+^

*N*-(3-Dimethylaminopropyl)-N'-ethylcarbodiimide hydrochloride (EDC), *N*-hydroxysuccinimide (NHS), copper (II) chloride (CuCl_2_), 1-hydroxybenzotriazole hydrate, and 2-(*N*-morpholino) ethanesulfonic acid (MES) were purchased from Sigma-Aldrich (Korea), and Silicon (Si) wafer (P-type) was obtained from Silicon Technology Corporation. Conductive signaling using atrogene-conjugated PD-Cu^2+^-coated electrodes with signaling factors. The signaling effects of atrogene-conjugated PD-Cu^2+^ and other signaling factors were investigated. The PD-Cu^2+^-coated electrode was prepared as previously described 17,18. To prepare the atrogene-conjugated PD-Cu^2+^-coated electrode, the PD-Cu^2+^-coated electrode was immersed in an EDC (2 mg/mL) solution for 30 min, and NHS (3 mg/mL) was added to the solution and stirred for 2 h. The PD-Cu^2+^-coated electrode was washed with water and the activated surface was dipped into an atrogenic (Trim63, Fbxo32 #1, and Fbxo32 #2) solution at a concentration 0.1 μg/mL for 12 h. The atrogene-conjugated PD-Cu^2+^-coated electrode obtained was washed with water and dried. To detect each factor, the surface was incubated with a solution of each factor [(miR-582-5p mimic and negative control miRNA) / (100 ng/mL)] for 12 h. The treated surfaces were washed with water and dried. To investigate electrochemical property, a 2-electrode system source meter, EIS (2-electrode system, 0.1-10^4^ Hz, 0 V vs OCP), and wireless system were employed. For cell transfection, GFP-tagging miR-582-5p mimic was transfected into HepG2 and differentiated C2C12 cells using Lipofectamine.

### Quantitative real-time (qRT)-PCR

Total RNA was isolated using RNAiso Plus (#9109; TaKaRa) according to the manufacturer's instructions. Then, 1 μg RNA was reverse-transcribed using the 5× All-In-One RT Master Mix (ABM, #G492). Real-time PCR was performed on an ABI StepOnePlus instrument (Applied Biosystems, Waltham, MA, USA) using the AMPIGENE qPCR Green Mix Hi-ROX (Enzo Biochem Inc., Farmingdale, NY, USA, ENZ-NUC103). The cycling protocol was 50 cycles of 95 °C for 10 s, 57 °C for 15 s, and 72 °C for 10 s. All qRT-PCR reactions were performed in triplicate. The relative expression level of each gene was normalized to that of 18S rRNA. The forward and reverse primer sequences are listed in Supplementary Table 1.

### Statistical analyses

In our experimental results, data are presented as mean ± standard deviation. The Student's t-test was used for data analysis, and statistical significance levels were denoted as follows: * P < 0.05, ** P < 0.01, *** P < 0.001, and **** P < 0.0001.

## Results

### The liver-muscle axis as a therapeutic target in NAFLD and sarcopenia

**Figure 1:**
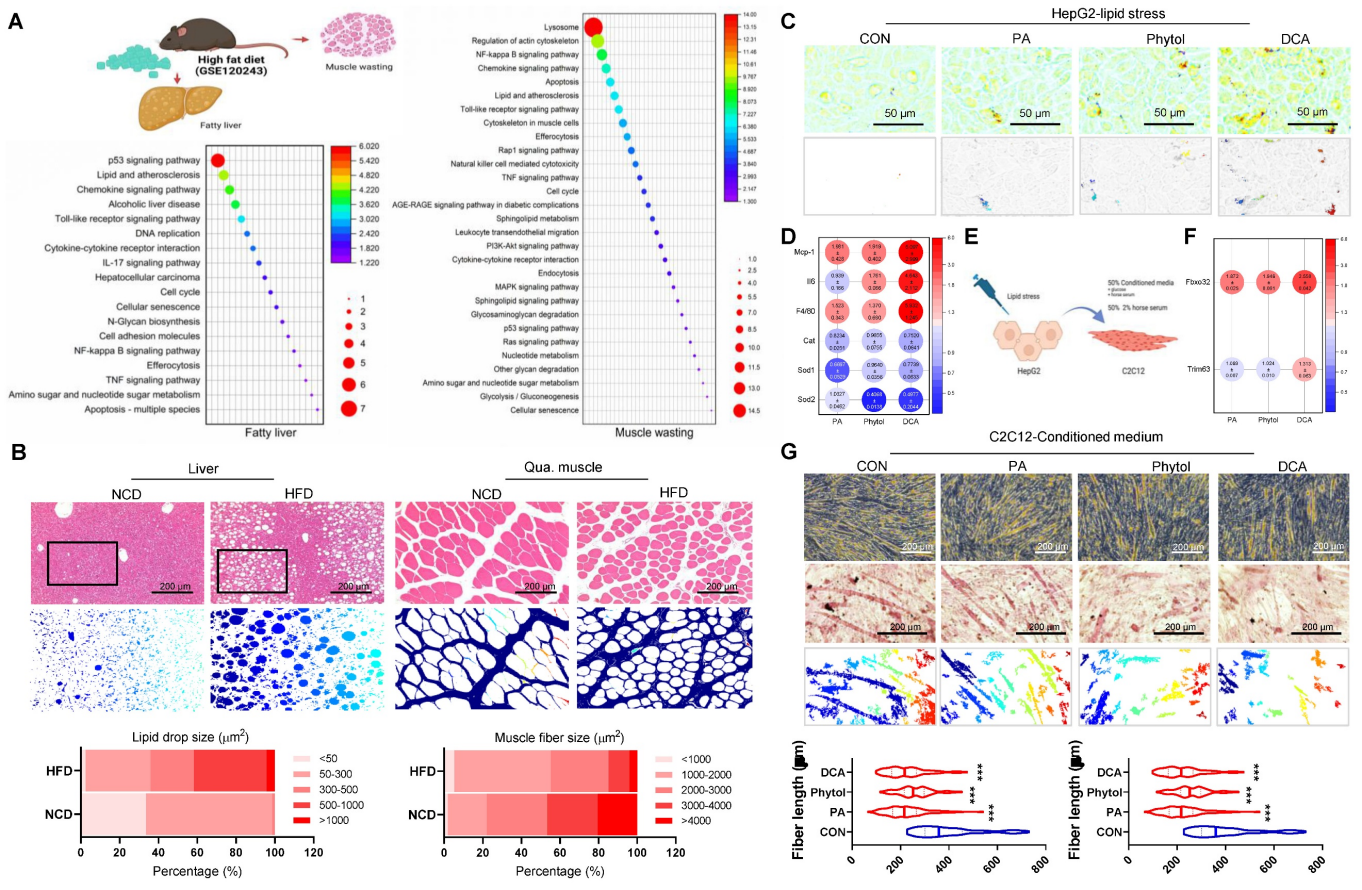
** Crosstalk between NAFLD and muscle wasting. (A)** Schematic representation of the liver-muscle axis in high-fat diet (HFD)-induced fatty liver and muscle wasting, along with pathway enrichment analysis. Significant upregulation of lipid metabolism and inflammatory pathways in liver and gastrocnemius muscle tissues is observed in HFD conditions (GSE120243, n = 3 mice/group)**. (B)** Histological analysis of liver and quadriceps muscle (Qua. muscle) tissues in mice fed on a normal chow diet (NCD) and HFD, showing lipid accumulation and muscle atrophy, respectively. Original magnification, 100 x, Scale bar = 200 μm. Liver lipid droplet sizes and quadriceps muscle fiber sizes were measured. **(C)** Oil red O staining in HepG2 cells treated with control (CON), palmitic acid (PA), phytol, and docosanoic acid (DCA). Original magnification, 200 x. **(D)** Expression profile of pro-inflammatory and antioxidant genes in HepG2 cells under different treatments. Results are presented as the mean ± standard deviation from three independent experiments (n = 3). **(E)** Schematic of conditioned medium (CM) transfer from HepG2 to C2C12 cells to simulate liver-muscle crosstalk under lipid stress.** (F)** Gene expression analysis of atrophy-related genes (*Fbxo32* and *Trim63*) in C2C12 cells treated with CM from lipid-stressed HepG2 cells. Results are presented as the mean ± standard deviation from three independent experiments (n = 3).** (G)** Representative images of myotube formation in C2C12 cells treated with CM from HepG2 cells under various conditions, indicating muscle atrophy. Original magnification, 200 x. Data is expressed as the mean ± SE, n = 3.

The liver-muscle axis plays a crucial role in the development of metabolic disorders, including NAFLD and sarcopenia. To investigate this crosstalk, we conducted an in silico analysis of sequencing data from NAFLD (GSE120243), followed by pathway enrichment analysis. The results revealed substantial upregulation of lipid metabolism dysfunction and inflammatory pathways in both the hepatic and skeletal muscle tissues of HFD mice (Figure 1A). This finding suggests that NAFLD-associated hepatic stress may lead to systemic inflammation and lipid dysregulation, impacting skeletal muscles and contributing to muscle atrophy.

The histological analysis further supported these observations. Liver tissues from HFD-fed mice exhibited significant lipid accumulation and structural disorganization compared to normal chow diet (NCD) controls (Figure 1B, left panel). Likewise, Qua. muscles from the HFD mice showed a marked decrease in myotube size, indicative of muscle wasting, which was absent in the NCD group (Figure 1B, right panel). Overall, these findings emphasize the potential role of NAFLD in promoting muscle atrophy through inflammatory and metabolic disturbances.

To further delineate the impact of liver-derived factors on muscle wasting, we treated HepG2 cells with lipotoxic agents, including PA, phytol, and docosanoic acid (DCA), which mimic the metabolic stress associated with NAFLD (Figure 1E). These treatments led to the upregulation of pro-inflammatory cytokines such as *Mcp-1*, *Il6*, and *F4/80* (Figure 1D), indicating a heightened inflammatory response. Moreover, antioxidant gene expression was downregulated (Figure 1D) and significant lipid accumulation and peroxidation were observed in the treated HepG2 cells, as evidenced by fluorescence microscopy (Figure 1C and Figure S1). This response suggests that NAFLD promotes an unfavorable environment that can affect other organs, including the skeletal muscle. When conditioned medium from stressed HepG2 cells was applied to C2C12 muscle cells, a notable increase in the expression of muscle atrophy-related genes *Fbxo32* and *Trim63* was observed (Figure 1F). Additionally, myotube formation was significantly impaired in C2C12 cells treated with this conditioned medium, as shown by morphological changes and decreased fiber density (Figure 1G). These results reinforce the hypothesis that liver-derived factors exacerbate muscle atrophy under NAFLD conditions, highlighting the liver-muscle axis as a potential therapeutic target for addressing both NAFLD and sarcopenia.

### Development and characterization of a ROS-sensitive MnO₂-PD (HA) hydrogel

**Figure 2:**
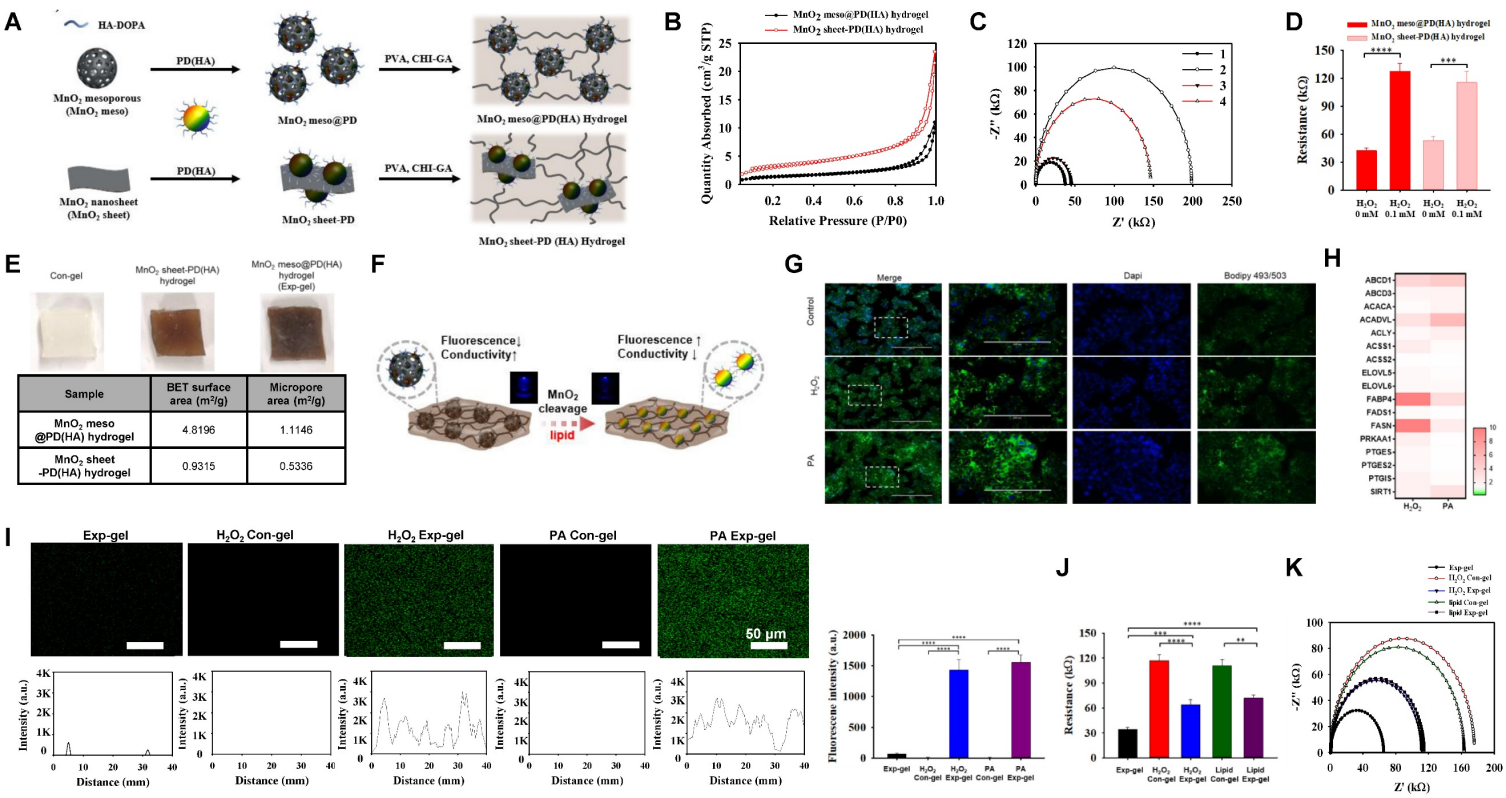
**Development and characterization of MnO_2_ mesoporous PD (HA) hydrogel (Exp-gel) for ROS-sensitive applications. (A)** Synthesis of Exp-gel via dopamine-conjugated hyaluronic acid (HA-DOPA) and polyvinyl alcohol (PVA)-CHI-GA complexes. **(B)** BET surface area analysis showing larger surface and micropore areas for Exp-gel compared to MnO_2_ nanosheet-PD (HA) hydrogel. **(C)** Nitrogen adsorption-desorption isotherm showing enhanced porosity in Exp-gel.** (D)** Resistance changes in hydrogels upon H_2_O_2_ treatment, indicating ROS-sensitive electrical response. n = 5. **(E)** Comparison of BET surface area and micropore area between MnO_2_ mesoporous and nanosheet hydrogels.** (F)** Schematic of ROS-triggered MnO_2_ degradation in Exp-gel, leading to fluorescence restoration and conductivity reduction. **(G)** Confocal images of ROS-triggered fluorescence recovery in Exp-gel with H_2_O_2_ and PA treatments. Original magnification, 200 x. Scale bar = 200 μm. n = 3.** (H)** Heatmap of lipid metabolism-related genes in HepG2 cells with ROS-sensitive Exp-gel.** (I-K)** Fluorescence and resistance analysis demonstrating dual detection of ROS through fluorescence recovery and conductivity changes in Exp-gel. Scale bar = 50 μm. Data is expressed as the mean ± SE. Fluorescence intensity profile of the Con-gel and Exp-gel treated with H_2_O_2_ or PA (n = 5). P values were determined using one-way ANOVA post hoc Bonferroni test: (**D, I and J**). ** P < 0.01, *** P <0.001, and **** P < 0.0001. Source data are provided as a Source Data file.

To address the liver-muscle crosstalk and its associated pathologies, we developed an ROS-sensitive Exp-gel, which was optimized for responsive signaling in the context of NAFLD and muscle wasting. MnO_2_ structures, including mesoporous and nanosheet forms, were incorporated into a dopamine-conjugated hyaluronic acid (PD (HA)) matrix to harness the shape-dependent properties of MnO_2_ for ROS-sensitive diagnostics. The fluorescence of PD (HA), which was quenched by the Förster resonance energy transfer (FRET), was restored following the degradation of MnO_2_ to Mn^2+^ by ROS, enabling dual fluorescence- and conductivity-based sensing (Figure 2A).

The Exp-gel exhibited a significantly larger BET surface area (4.8196 m²/g) and micropore area (1.1146 m²/g) than the MnO_2_ nanosheet-PD (HA) hydrogel, indicating its superior porosity (Figure 2B). EIS showed greater sensitivity to H_2_O_2_ for the mesoporous hydrogel, which exhibited a substantial decrease in conductivity upon the ROS-induced MnO_2_ decomposition (Figures 2C-E). The DPPH inhibition effect (83.1%), and hydroxyl radical scavenging activity (78.1%) of the Exp-gel, demonstrated excellent antioxidant properties compared to the control hydrogel (Figure S2A). Additionally, the structural advantage of Exp-gel allowed mesoporous hydrogel to interact more effectively with ROS, leading to enhanced fluorescence restoration and conductivity reduction compared to the nanosheet hydrogel (Figure 2F, Figure S2B, and S2C). These properties make Exp-gel an ideal candidate for detecting ROS levels in environments associated with oxidative stress such as NAFLD. Exp-gel hydrogel exhibited viscoelastic behavior (G' > G''), with H₂O₂ treatment reducing the strain crossover from 16.4 % to 6.2 %, and the incorporation of the gallic acid moiety in CHI-GA enhanced its adhesive properties on substrates including rubber, plastic, glass, and metal (Figure S2D and S2E). Compression tests further revealed that H_2_O_2_ treatment enhanced the elasticity of the hydrogel, as evidenced by an increase in the maximum stress from 0.026 MPa (control) to 0.033 MPa (H_2_O_2_ -treated) (Figure S2F). This mechanical stability under oxidative conditions suggests that the hydrogel retains its structural integrity in an inflamed tissue environment.

### Dual diagnostic and therapeutic effects of MnO_2_-PD (HA) hydrogel in modulating liver-muscle crosstalk

**Figure 3:**
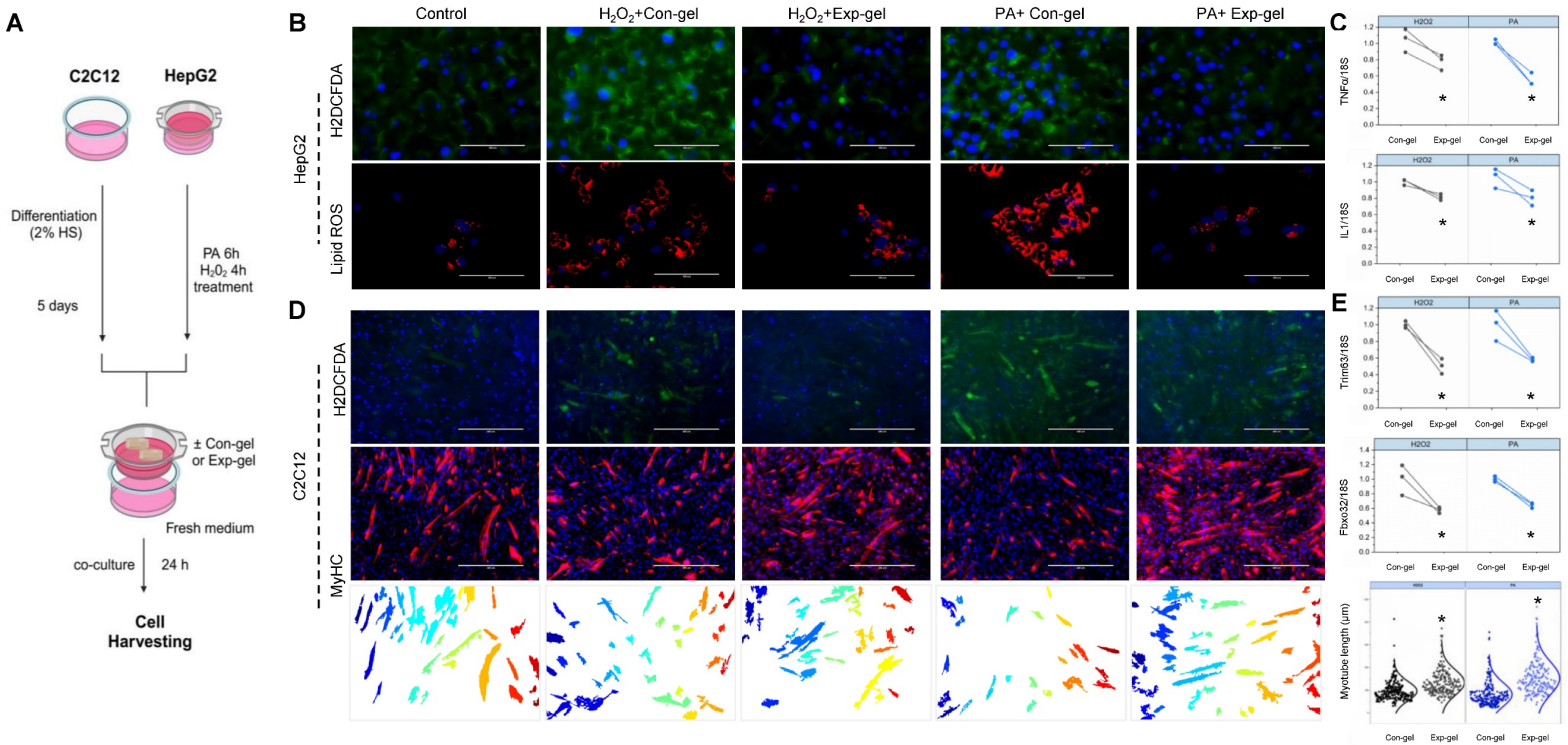
**Effects of Exp-gel on ROS levels and atrophy markers in HepG2-C2C12 co-culture. (A)** Schematic representation of the in vitro experimental design. **(B)** Confocal imaging of HepG2 cells showing ROS and lipid ROS levels under H_2_O_2_ and PA treatments with Con-gel and Exp-gel treatments. Original magnification, 100x. Scale bar = 100 μm. **(C)** Quantification of pro-inflammatory cytokine expression (*Il1* and *TNFα*) in HepG2 cells with different treatments, n = 3. **(D)** Representative images of myotube formation in C2C12 cells co-cultured with stressed HepG2 cells and treated with Con-gel or Exp-gel, showing protective effects of Exp-gel on muscle cells. Original magnification, 400 x. Scale bar = 400 μm. **(E)** Gene expression analysis of atrophy markers (*Trim63* and *Fbxo32*) in C2C12 cells under different treatment conditions, n = 3. Data is expressed as the mean ± SE. P values were determined using unpaired two-tailed Student′s t-test: (**C, E**). * p < 0.05. Source data are provided as a Source Data file.

HepG2 cells cultured with hydrogels at varying doses and NP percentages exhibited no toxicity, allowing for the identification of an optimal in vitro dose (Figure S3), and subsequent co-culture experiments with C2C12 cells using Exp-gel or Con-gel were conducted to assess the hydrogel's impact on liver-muscle crosstalk. In HepG2 cells treated with H_2_O_2_ or PA, the hydrogel exhibited fluorescence and electrical responses indicative of MnO_2_ degradation owing to elevated ROS (Figures 2G-I). The ROS-induced fluorescence recovery and increased electrical resistance in the Exp-gel compared to those in the Con-gel underscores its ability to detect and respond to oxidative stress (Figures 2I-K). Additionally, Exp-gel treatment in this co-culture system mitigated ROS accumulation and pro-inflammatory cytokine expression (*Il1* and *TNFα*) in HepG2 cells (Figures 3A-C), thereby reducing muscle atrophy markers in C2C12 cells.

Furthermore, the co-culture demonstrated that Exp-gel preserved the myotube structure and minimized muscle degradation in C2C12 cells exposed to stressed HepG2 cells, as evidenced by the reduced ROS accumulation and improved myotube alignment (Figures 3D and 3E). Exp-gel significantly attenuated H_2_O_2_- and palmitic acid (PA)-induced oxidative stress in HepG2 cells, as shown by the decreased H2DCFDA fluorescence and lipid ROS levels (Figures 3B). This protective effect was further translated into C2C12 cells, where the Exp-gel reduced oxidative damage and enhanced myotube integrity compared to the Con-gel. In conditioned medium experiments, Exp-gel effectively attenuated the deleterious effects of inflammatory liver secretions on muscle cells. HepG2 cells treated with Exp-gel secreted factors that promoted robust myotube formation and reduced myotube degradation in C2C12 cells (Figure S4). Quantitative analysis confirmed a significant improvement in markers of muscle health, such as *Fbxo32* and *Trim63*, in the Exp-gel-treated group compared to those for Con-gel under both normal and stressed conditions. These results suggest that Exp-gel not only protects against oxidative stress in hepatocytes but also mitigates liver-muscle signaling disturbances, highlighting its potential therapeutic utility in combating muscle degradation associated with NAFLD.

### In vivo evaluation of exp-gel in HFD-induced NAFLD and muscle atrophy

**Figure 4:**
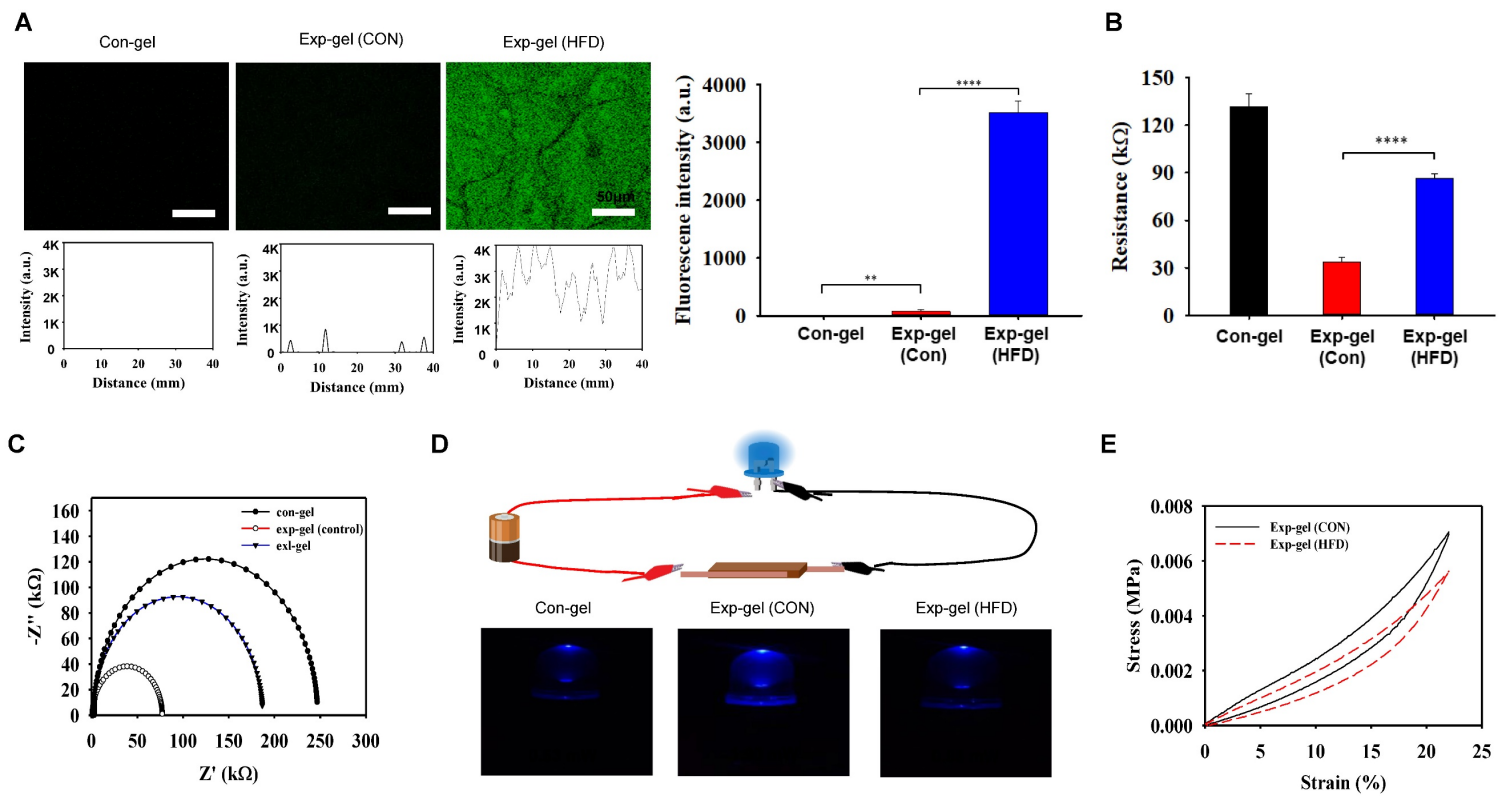
**In vivo efficacy of Exp-gel in an HFD-induced NAFLD model. (A)** Fluorescence imaging of Exp-gel in HFD mice, showing ROS-triggered fluorescence recovery. Scale bar = 50 μm. Fluorescence intensity profile of the Con-gel, Exp-gel (Con), and Exp-gel (HFD) (n = 5). **(B** and **C)** EIS profile indicating reduced conductivity in Exp-gel under HFD conditions. Sourcemeter analysis showing increased resistance in Exp-gel in response to HFD-induced ROS, n = 5. **(D)** Schematic of LED-based electrical evaluation showing light intensity changes due to ROS-sensitive MnO_2_ degradation in Exp-gel, and images of the Con-gel, MnO_2_ sheet-PD (HA) hydrogel, and MnO_2_ meso@PD (HA) hydrogel (Exp-gel). **(E)** Compression strength (0-23 % strain) of Exp-gel treated with and without HFD. Data is expressed as the mean ± SE. P values were determined using one-way ANOVA post hoc Bonferroni test: (**A, B**). ** P < 0.01, and **** P < 0.0001. Source data are provided as a Source Data file.

To evaluate Exp-gel's in vivo efficacy, HFD-fed mice were implanted with either Exp-gel or Con-gel in the liver, following a four-week subcutaneous implantation that confirmed its long-term biocompatibility with minimal immune response via F4/80 staining (Figure S5). The Exp-gel exhibited restored fluorescence owing to MnO_2_ reduction in the high-ROS environment of the HFD model, indicating ROS-sensitive activation (Figure 4A). EIS profiles showed reduced conductivity in the Exp-gel under HFD conditions, which was attributed to MnO_2_ cleavage. Further, source meter analysis confirmed an increase in resistance, reinforcing its ROS responsiveness (Figures 4B and 4C). Source meter resistance analysis revealed increased resistance from 33.9 kΩ in control hydrogels to 87.1 kΩ in Exp-gel-implanted HFD mice, confirming a strong response to the oxidative environment. Furthermore, LED-based evaluations revealed that Exp-gel exhibited ROS-sensitive changes in light intensity—a feature absent in Con-gel (Figure 4D). Additionally, compression tests in the HFD model demonstrated that Exp-gel enhanced the hydrogel's elasticity through MnO₂ reduction to Mn²⁺ via ROS scavenging, serving as a distinctive marker for high ROS levels (Figure 4E). Body weight, liver weight relative to body weight, and skeletal muscle weight relative to body weight showed no changes in Exp-gel-HFD mice compared to those in Con-gel-treated mice (Figure S6).

**Figure 5:**
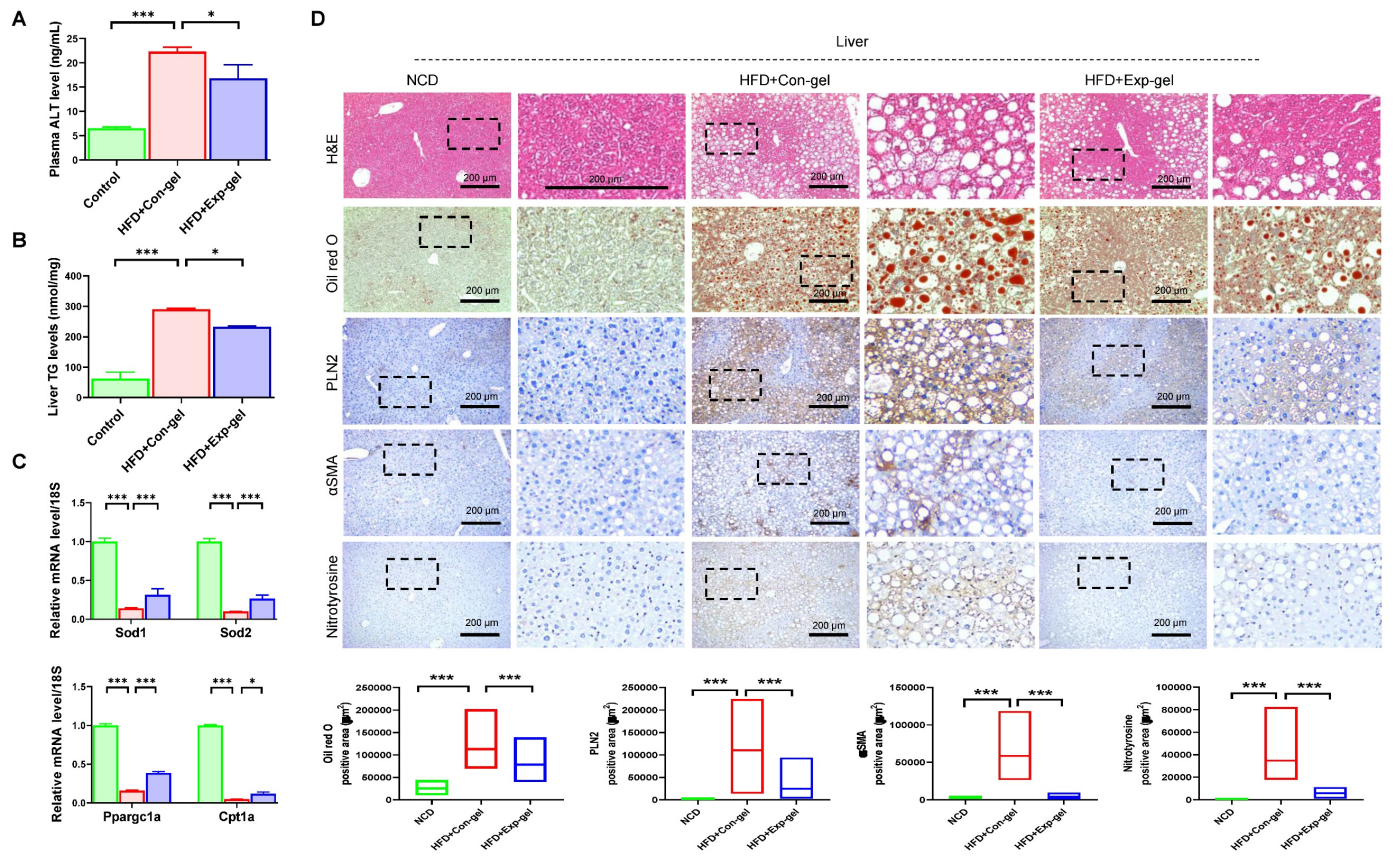
**Histological analysis of liver in HFD mice treated with Exp-gel. (A)** Plasma ALT level. n = 3. **(B)** Liver TG levels. n = 3. **(C)** Quantification of *Sod1*, *Sod2*, *Ppargc1a*, and *Cpt1a* in liver. n = 3. **(D)** Representative images of liver sections stained with H&E, Oil red O, PLIN2, αSMA, and nitrotyrosine in NCD, HFD-Con-gel, and HFD-Exp-gel groups. Exp-gel treatment significantly reduced lipid accumulation, fibrosis markers (αSMA), and ROS-related damage (nitrotyrosine), suggesting protective effects on liver tissue architecture in an HFD environment. Original magnification, 200 x. Scale bar = 200 μm. Data is expressed as the mean ± SE of three mice per group. P values were determined using one-way ANOVA post hoc Bonferroni test: (**A, B, C, D**). * P < 0.05 and *** P < 0.001. Source data are provided as a Source Data file.

Exp-gel implantation significantly reduced plasma ALT and liver TG levels (Figure 5A and 5B). Moreover, it enhanced the expression of key antioxidant genes (*Sod1* and *Sod2*) and improved markers of mitochondrial function (*Ppargc1a* and *Cpt1a*) in the liver (Figure 5C). Histological analysis revealed that Exp-gel implantation in the HFD-fed mice significantly mitigated hepatic damage. Specifically, Exp-gel reduced ROS accumulation, as evidenced by decreased nitrotyrosine staining, minimized fibrosis through reduced αSMA staining, and restored liver architecture, including decreased steatosis and inflammation, compared to the Con-gel controls (Figure 5D). Quantitative analyses further confirmed the reduced levels of oxidative stress markers and fibrosis-related gene expression in the Exp-gel group, underscoring its therapeutic potential in alleviating hepatic inflammation and structural damage (Figure S6 and Figure 5D).

**Figure 6:**
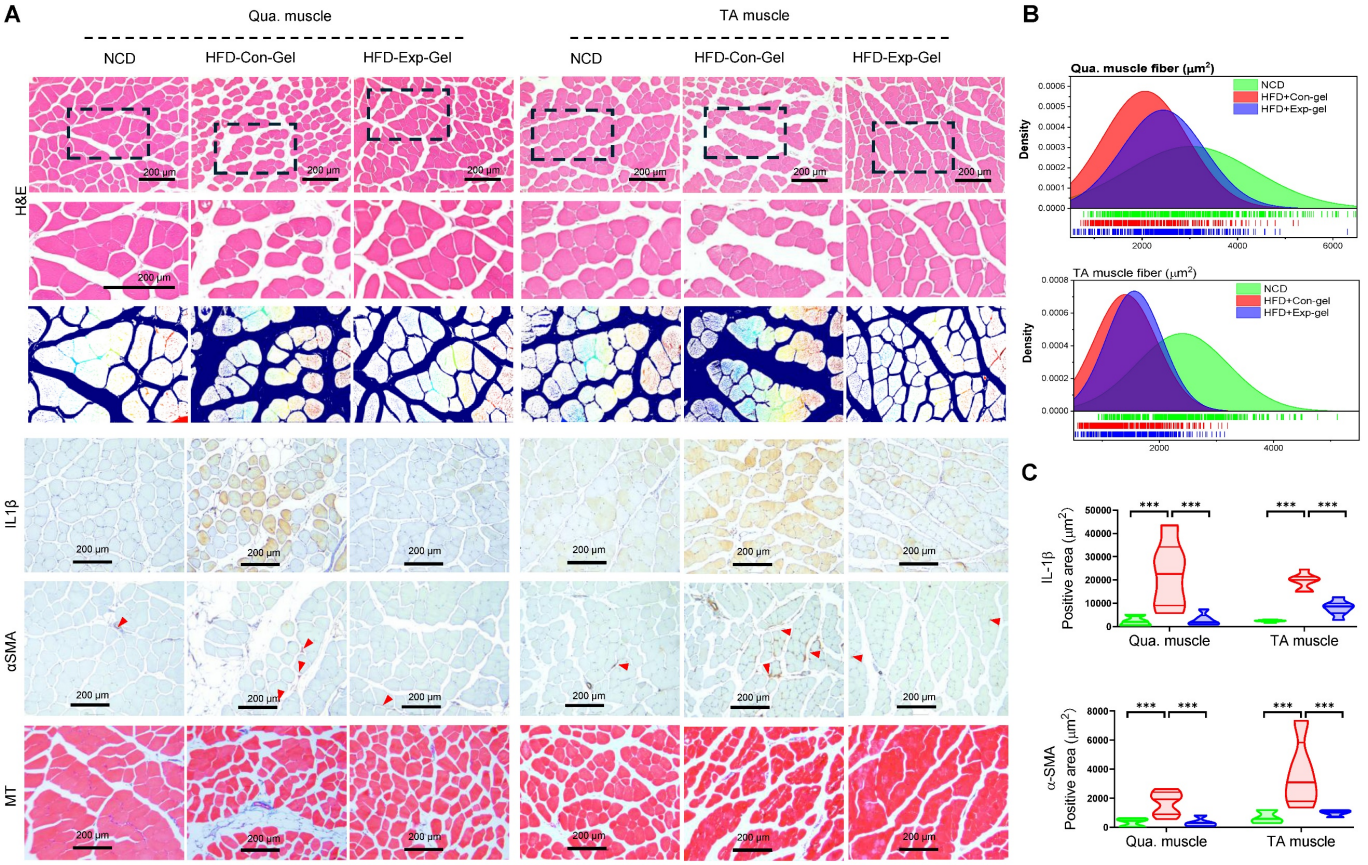
** Histological analysis of quadriceps muscle tissues in HFD mice treated with Exp-gel. (A)** Representative images of H&E staining, Masson's trichrome staining, and immunohistochemistry (IHC) for IL-1β and α-SMA in quadriceps (Qua.) and tibialis anterior (TA) muscle sections from normal chow diet (NCD), HFD-Con-gel, and HFD-Exp-gel groups. **(B)** Muscle fiber size distribution analysis in Qua. and TA muscles. **(C)** Quantification of IL-1β and α-SMA positive areas using ImagePro software. Images were captured at 200 x magnification (scale bar = 200 μm). Data is presented as mean ± SE for three mice per group. Statistical significance was determined using one-way ANOVA followed by Bonferroni post hoc test (*** P < 0.001). Source data are provided as a Source Data file.

**Figure 7:**
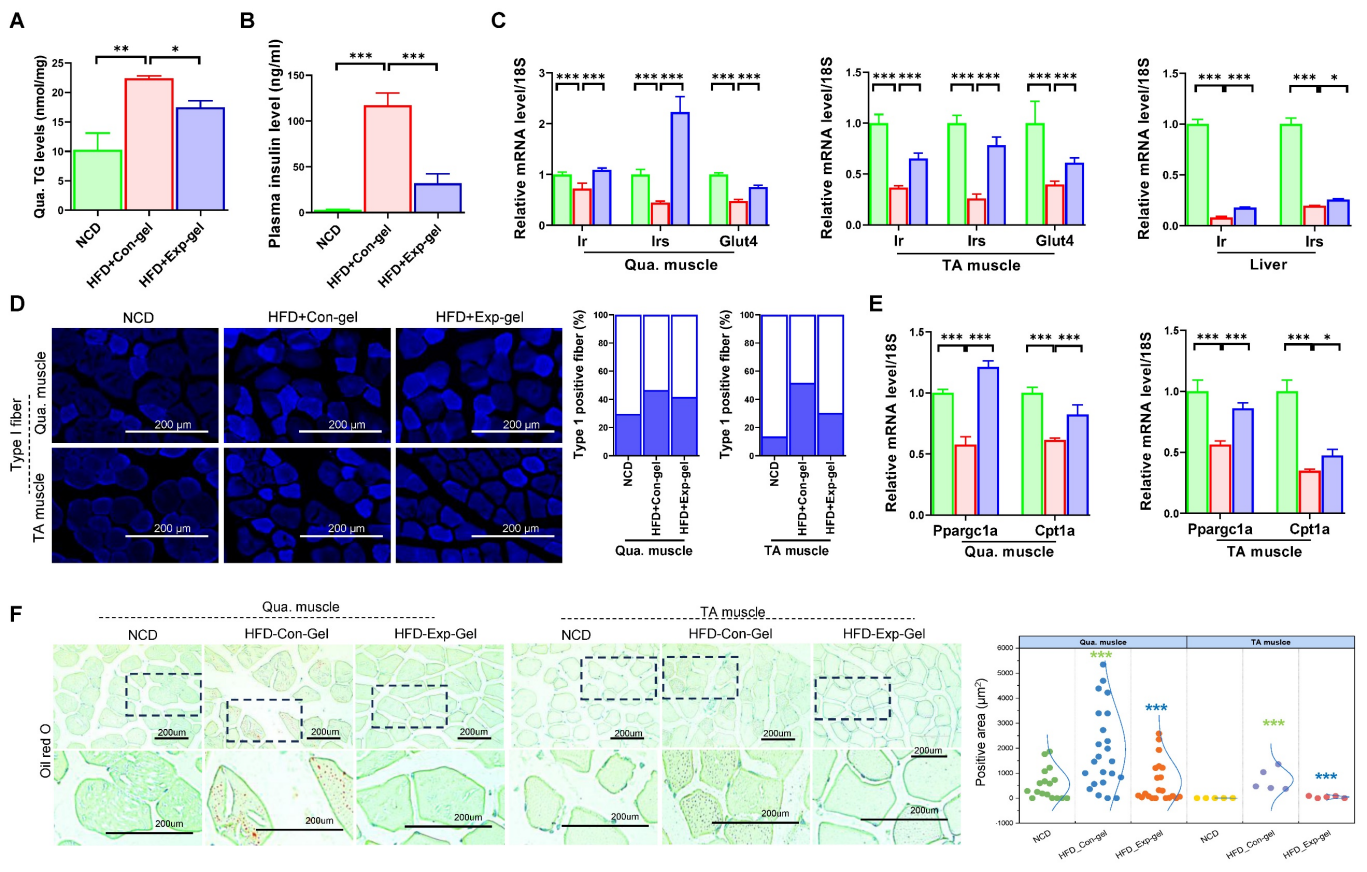
**Effects of Exp-gel on insulin resistance. (A)** Hepatic triglyceride (TG) levels in liver tissue (n = 3). **(B** and** C)** Plasma ALT levels (n = 3) and quantification of insulin signaling-related genes (*Ir*, *Irs*, and *Glut4*) in quadriceps (Qua.), tibialis anterior (TA) muscle, and liver tissues. **(D)** Immunofluorescence (IF) staining of Type 1 muscle fibers, with quantification of positive fibers. **(E)** Expression levels of insulin signaling-related genes (*Ppargc1a* and *Cpt1a*) in Qua. and TA muscles. **(F)** Oil Red O staining of liver tissue to assess lipid accumulation. Data is presented as mean ± SE for three mice per group. Statistical significance was determined using one-way ANOVA followed by Bonferroni post hoc test (* P < 0.05, ** P < 0.01, and *** P < 0.001). Source data are provided as a Source Data file.

Moreover, morphological evaluation and histological quantification demonstrated improved muscle fiber integrity and reduced inflammation and fibrosis in the Exp-gel group than in the Con-gel group (Figure 6). Exp-gel improved TG levels in the Qua. muscle, and significantly decreased plasma insulin level (Figure 7A and 7B). Moreover, the expressions of *Ir*, *Irs*, and *Glut4* were markedly upregulated in the Qua muscle, TA muscle, and liver (Figure 7C). Although long-term HFD increased type I muscle fiber content, it decreased the expression of mitochondrial markers *Ppargc1a* and *Cpt1a*—indicating impaired mitochondrial function (Figures 7D and 7E). Notably, Exp-gel treatment enhanced mitochondrial function in muscle. Additionally, Exp-gel effectively inhibited lipid infiltration in both Qua. and TA muscles. This suggests that the hepatic improvements achieved by Exp-gel indirectly alleviated muscle wasting, mitochondria dysfunction, and lipid accumulation associated with NAFLD. These results highlight the dual role of Exp-gel in improving hepatic health and mitigating NAFLD-associated myopathies, thus offering a promising therapeutic strategy for systemic metabolic disorders.

### miR-582-5p-mediated modulation of liver-muscle crosstalk by MnO_2_-PD (HA) hydrogel

**Figure 8:**
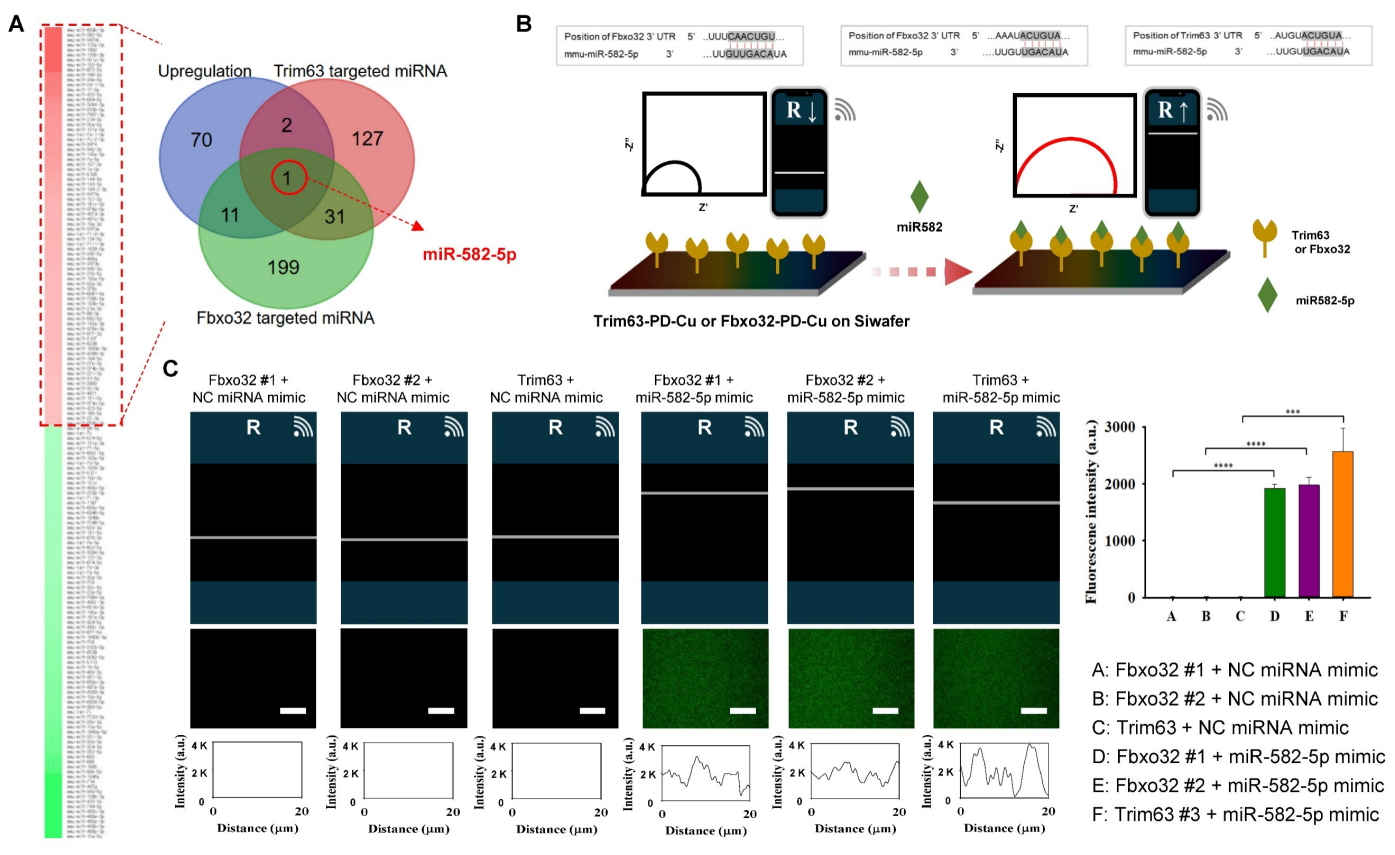
**Exosomal miRNA profiling in HFD mice treated with Exp-gel. (A)** Schematic of exosome isolation and miRNA sequencing from liver tissue of HFD mice implanted with Exp-gel. n = 3.** (B)** Heatmap showing upregulation of miR-582-5p in exosomes derived from HFD liver implanted with Exp-gel, highlighting its role in modulating atrophy genes *Fbxo32* and Trim63.** (C)** Electrochemical impedance spectroscopy (EIS) confirming miR-582-5p's interaction with atrophy-related targets. Fluorescence intensity profile of the signaling the atrogene-conjugated PD-Cu^2+^ coated electrode with signaling factors. Increased resistance measurements further validate the therapeutic effect of miR-582-5p on atrogenic gene inhibition. Scale bar = 50 μm. Data is expressed as the mean ± SE for three mice per group, with P values determined using one-way ANOVA followed by a Bonferroni post hoc test (**** P < 0.0001).

To explore the mechanistic basis of the protective effects of Exp-gel, we analyzed exosomal miRNA profiles in HFD-fed mice implanted with Exp-gel. Dynamic light scattering analysis revealed that the isolated exosomes had an average size of 134.2 nm, and western blot confirmed their success isolation by detecting the exosome-specific markers CD81 and TSG101 (Figure S7). miRNA sequencing revealed significant upregulation of miR-582-5p, a key regulator of the atrogenic genes Fbxo32 and Trim63 (Figure 8A). Pathway analysis further identified miR-582-5p as a modulator of several pathways crucial for muscle integrity, including ubiquitin-mediated proteolysis and TGF-β signaling, underscoring its therapeutic potential in modulating liver-muscle crosstalk through miRNA-based exosomal delivery (Figure S8). Moreover, Exp-gel treatment upregulates a network of miRNAs (Supplementary Table 2)—including 11 that downregulate Fbxo32 and 2 that downregulate Trim63—enriching pathways in inflammation, TGFβ signaling, lipid metabolism, and ubiquitin‑regulated proteolysis to collectively promote muscle homeostasis, with miR-582-5p playing a key role (Figure S8).

To evaluate the interaction between miR-582-5p and the atrogenic genes (*Fbxo32* and *Trim63*), we employed EIS using a PD-Cu^2+-^coated electrode system (Figure 8B). The conjugation of the atrogene 3'UTRs (*Fbxo32-*3'UTR and *Trim63-*3'UTR) to the PD-Cu^2+^ electrode increased the resistance, reflecting the successful immobilization of target genes on the electrode surface. When treated with the negative control (NC) miRNA mimic, no significant changes in resistance were observed, confirming the lack of interaction between the negative control and atrogene-conjugated electrodes. In contrast, treatment with the miR-582-5p mimic induced a significant increase in the resistance of electrodes conjugated with Fbxo32 and Trim63, indicating specific and selective interactions between miR-582-5p and these atrogenic gene targets. Additionally, a fluorescence-labeled miR-582-5p mimic was used to validate these interactions by confocal laser scanning microscopy (CLSM). Consistent with the EIS results, strong fluorescence signals were observed on PD-Cu^2+^ electrodes conjugated with *Fbxo32-*3'UTR and *Trim63-*3'UTR after miR-582-5p treatment, whereas minimal fluorescence was detected in NC miRNA-treated groups. These findings confirmed the selective binding of miR-582-5p to atrogenic gene targets (Figure 8C and Figure S9). Combined electrochemical and fluorescence-based approaches demonstrated that miR-582-5p interacts specifically and selectively with Fbxo32 and Trim63, further supporting its role in regulating osteogenic signaling pathways and contributing to the therapeutic effects of Exp-gel.

**Figure 9:**
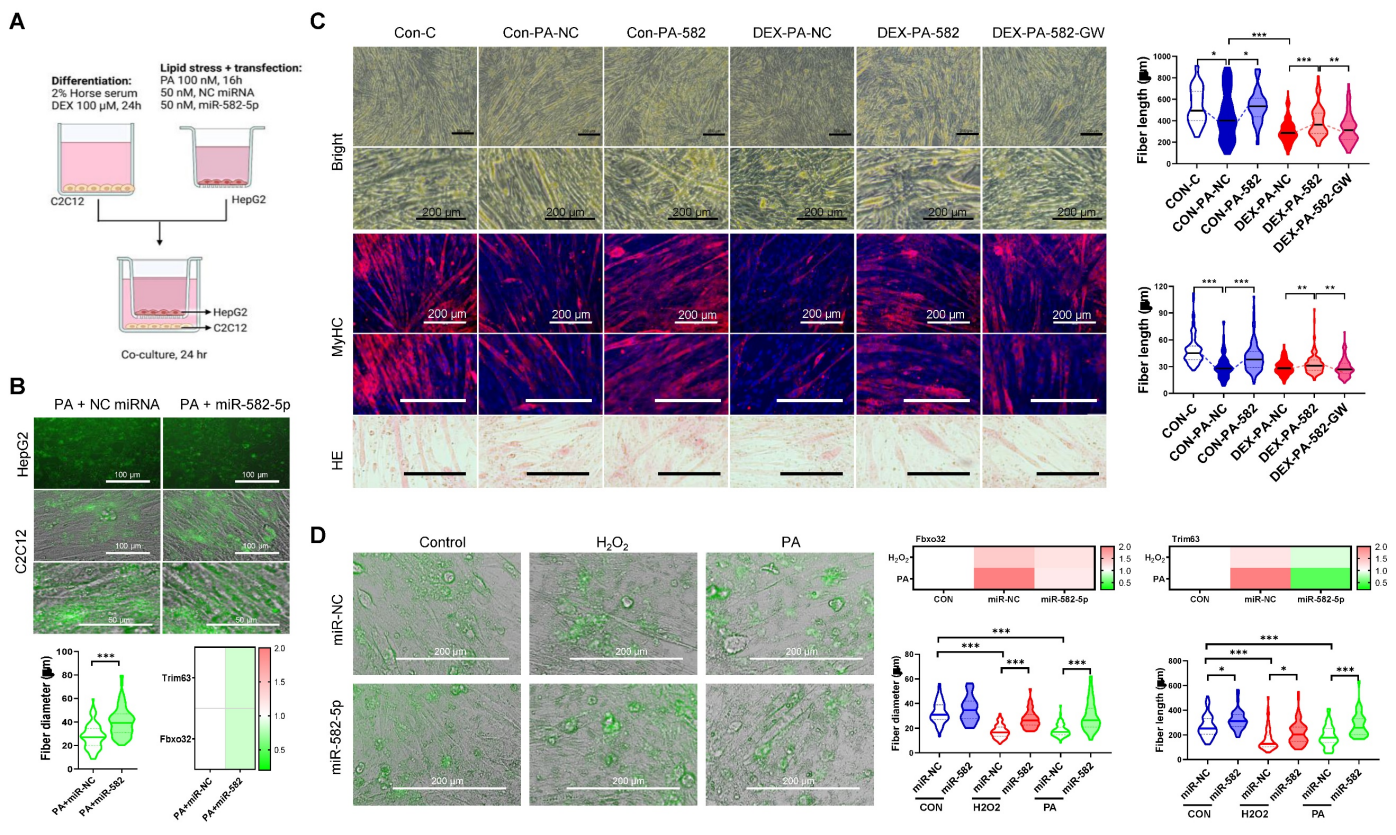
**Functional validation of miR-582-5p in liver-muscle crosstalk. (A)** Co-culture system shows exosomal transfer of GFP-tagged miR-582-5p from HepG2 cells to C2C12 cells. Original magnification of HepG2, 200 x. Scale bar = 200 μm; original magnification of C2C12, 100 x. Scale bar = 100 μm. n = 3, * P < 0.05. **(B, C)** Fluorescence images and myotube formation analysis in C2C12 cells, showing inhibition of atrophy pathways with miR-582-5p treatment. Original magnification, 100 x and 200 x, Scale bar = 200 μm. n = 3, * P < 0.05, ** P < 0.01, and *** P < 0.001.** (D)** Quantitative analysis of myotube size and gene expression of atrophy markers in C2C12 cells after miR-582-5p transfer, demonstrating the protective effect of miR-582-5p against muscle degradation in response to oxidative stress. Original magnification, 200 x, Scale bar = 200 μm. Data is expressed as the mean ± SE of three sets. P values were determined using one-way ANOVA post hoc Bonferroni test (* P < 0.05 and *** P < 0.001, n = 3).

Further functional in vitro studies revealed that exosomal miR-582-5p transfer from HepG2 cells to C2C12 myoblasts reduced the activation of atrogenic pathways and preserved myotube integrity under oxidative stress. C2C12 cells treated with the miR-582-5p mimic exhibited decreased expression of *Fbxo32* and *Trim63*, along with enhanced myotube formation, compared to the NC-treated controls, even under PA- or H_2_O_2_-induced stress (Figures 9A-D). Additionally, dexamethasone (DEX)-, H_2_O_2_-, and PA-induced muscle atrophy models demonstrated that miR-582-5p treatment improved muscle fiber size and alignment, further supporting its protective effects (Figure 9C and 9D). Collectively, these results highlight miR-582-5p as a central mediator in the therapeutic action of Exp-gel, acting to restore liver-muscle homeostasis by suppressing atrogenic signaling and preserving muscle integrity.

## Discussion

This study demonstrates the therapeutic potential of MnO_2_ Exp-gel in simultaneously addressing the dual pathologies of NAFLD and sarcopenia by modulating the liver-muscle axis. Our findings highlight the utility of an innovative ROS-sensitive hydrogel system for mitigating hepatic and muscular dysfunctions by targeting oxidative stress and inflammation, two critical mediators of the interplay between these conditions. This study also identified exosomal miR-582-5p as a pivotal mediator of liver-muscle crosstalk, adding a novel layer of understanding to the mechanisms underlying NAFLD-associated muscle wasting. Our results underscore the critical role of oxidative stress in NAFLD progression and its systemic effects on skeletal muscles. Elevated ROS levels in the liver drive hepatocyte injury, inflammation, and fibrosis, creating a proinflammatory milieu that disrupts muscle metabolism and promotes atrophy. Similarly, muscle atrophy is characterized by mitochondrial dysfunction and the activation of atrogenic pathways, both of which are exacerbated by oxidative stress. Given these shared pathophysiological underpinnings, therapeutic strategies aimed at ROS scavenging are promising.

The Exp-gel demonstrated robust ROS-scavenging ability, as evidenced by its fluorescence and conductivity responses in both in vitro and in vivo models. This dual diagnostic and therapeutic capacity is advantageous in conditions such as NAFLD and sarcopenia, where the dynamic monitoring of oxidative stress could guide treatment strategies. Furthermore, the ability of the hydrogel to preserve muscle fiber integrity and mitigate liver fibrosis in HFD-fed mice emphasized its therapeutic efficacy in breaking the detrimental cycle of the liver-muscle axis.

A particularly compelling finding of this study was the identification of miR-582-5p as a mediator of the therapeutic effects of Exp-gel. Exosomal miR-582-5p was upregulated in Exp-gel-treated HFD mice and was shown to modulate atrogenic gene expression in skeletal muscles. Previous studies have implicated exosomal miRNAs in interorgan communication; however, this study provides novel evidence for miR-582-5p as a potential therapeutic target. Downregulation of atrogenic genes, such as Fbxo32 and Trim63, by miR-582-5p underscores its protective role against muscle atrophy. This aligns with the findings from co-culture and transfection experiments, where miR-582-5p attenuated oxidative stress-induced muscle damage. By elucidating this mechanism, our study provides a foundation for exploring miRNA-based therapies for the treatment of metabolic disorders.

The clinical significance of our findings lies in the potential of Exp-gel to address two interrelated and prevalent conditions, NAFLD and sarcopenia, that pose significant healthcare burdens, particularly in aging populations. Current therapies focus on lifestyle changes or symptom management and fail to address the systemic interplay between these conditions. Exp-gel offers a dual-targeting approach that reduces hepatic oxidative stress, inhibits fibrogenesis, and preserves muscle integrity. Its ROS-responsive properties enable real-time monitoring and personalized therapeutic intervention, thereby enhancing its clinical applicability. This study also emphasizes the broad potential of nanotechnology for treating multifactorial diseases. The modular design of Exp-gel, which integrates ROS-sensitive diagnostics with therapeutic functions, demonstrates the versatility of MnO_2_ nanoparticles in oxidative stress modulation. Beyond NAFLD and sarcopenia, these properties could extend to other oxidative stress-related conditions such as cardiovascular diseases, neurodegenerative disorders, and cancer. Incorporating exosomal delivery systems further enhances targeted therapeutic strategies, paving the way for innovative treatments for complex metabolic diseases.

## Conclusion

This study provides compelling evidence for the therapeutic potential of MnO_2_ mesoporous PD (HA) hydrogels in addressing the interconnected pathologies of NAFLD and sarcopenia. Exp-gel is a novel and effective approach for treating these conditions by targeting oxidative stress and modulating the liver-muscle axis through exosomal miR-582-5p. Its dual diagnostic and therapeutic properties, combined with mechanistic insights into miRNA-mediated interorgan communication, pave the way for innovative strategies for the management of metabolic diseases. Clinical translation of this technology has far-reaching implications, offering a comprehensive solution to the multifactorial challenges posed by NAFLD and sarcopenia. Future studies should focus on optimizing hydrogel formulation, evaluating its long-term safety, and conducting clinical trials to validate its efficacy in humans. With continued advancements in nanotechnology and miRNA therapeutics, the Exp-gel platform has the potential to revolutionize the treatment of complex metabolic disorders.

## Supplementary Material

Supplementary figures and tables.
