# Supplementary material for: ROS-responsive MnO2 mesoporous hydrogel to modulate liver-muscle crosstalk and mitigate NAFLD-associated sarcopenia via exosomal miR-582-5p delivery
Source: Theranostics. 2025 Mar 21;15(10):4579–92. doi: 10.7150/thno.108280 (PMC11984410; doi:10.7150/thno.108280)
Supplement: Supplementary file 1 — Supplementary figures and tables. [file thnov15p4579s1.pdf]

Supplemental Table 1. List of Primers used in this study

Gene symbol	Forward	Reverse
RN18S	CCAGTAAGTGCGGGTCATAAG	GGCCTCACTAAACCATCCAA
Fbxo32	GTCGCAGCCAAGAAGAGAAAGA	TGCTATCAGCTCCAACAGCCTT
Cat	GATGGTAACTGGGATCTTGTGG	GTGGGTTTCTCTTCTGGCTATG
F4/80	TGTACGTGCAACTCAGGACT	GTGGGACCACAGAGAGTTGA
Glut4	AAAAGTGCCTGAAACCAGAG	TCACCTCCTGCTCTAAAAGG
Il1	GTCAACGTGTGGGGGATGAA	AAGCAATGTGCTGGTGCTTC
Il6	AGTTGCCTTCTTGGGACTGA	TCCACGATTTCCCAGAGAAC
Ir	AAATGCAGGAACTCTCGGAAG CCT	ACCTTCGAGGATTTGGCAGACCTT
Irs	ATGGGTACATGAGCATGGATAG	CAGGCGTGGTTAGGGAATAA
Mcp-1	TTAAAAACCTGGATCGGAACCAA	GCATTAGCTTCAGATTTACGGGT
Ppargc1a	CTAGCCATGGATGGCCTATTT	GTCTCGACACGGAGAGTTAAAG
Sod1	CTCAGGAGAGCATTCCATCATT	CTCCCAGCATTTCCAGTCTT
Sod2	AGCGTGACTTTGGGTCTTT	AGCGACCTTGCTCCTTATTG
Trim63	TAACTGCATCTCCATGCTGGTG	TGGCGTAGAGGGTGTCAAACTT
Tnfa	TTGTCTACTCCCAGGTTCTCT	GAGGTTGACTTTCTCCTGGTATG

Supplemental Table 2. Upregulated exosomal miRNA from liver with Exp-gel treatment

Fbxo32 target miRNA	11	miR-31-5p	miR-374c-5p	miR-345-5p
		miR-342-3p	miR-1839-5p	miR-185-5p
		miR-150-5p	miR-374b-5p	miR-345-3p
		miR-151-3p	miR-214-3p	
Trim63 target miRNA	2	miR-664-5p	miR-1892	
Others	70	let-7f-2-3p	miR-92a-3p	miR-139-5p
		miR-3473a	miR-22-3p	miR-3470b
		let-7c-2-3p	miR-3068-3p	miR-17-5p
		miR-27b-5p	miR-200b-5p	miR-30a-5p
		miR-872-5p	miR-1a-3p	miR-669b-3p
		miR-24-1-5p	miR-1843b-3p	miR-423-5p
		miR-130b-3p	miR-98-3p	let-7a-1-3p
		miR-3473e	miR-6981-5p	miR-3084-3p
		miR-151-5p	miR-142a-3p	miR-467a-3p
		miR-144-5p	miR-5126	miR-2137
		miR-425-5p	miR-7667-3p	miR-145a-5p
		miR-195a-5p	miR-669h-3p	miR-3960
		miR-6238	miR-186-3p	miR-378a-3p
		miR-378c	miR-101a-5p	miR-143-3p
		miR-19a-3p	miR-21a-3p	miR-467f
		miR-194-5p	miR-34a-5p	miR-677-3p
		miR-3474	miR-301a-3p	miR-7085-5p
		miR-27b-3p	miR-125a-5p	miR-221-3p
		miR-194-2-3p	miR-466q	miR-107-3p
		miR-467d-3p	miR-181c-5p	miR-378a-5p
		miR-382-5p	miR-3473b	let-7f-1-3p
		miR-32-3p	miR-193b-5p	miR-7a-5p

Figure legends

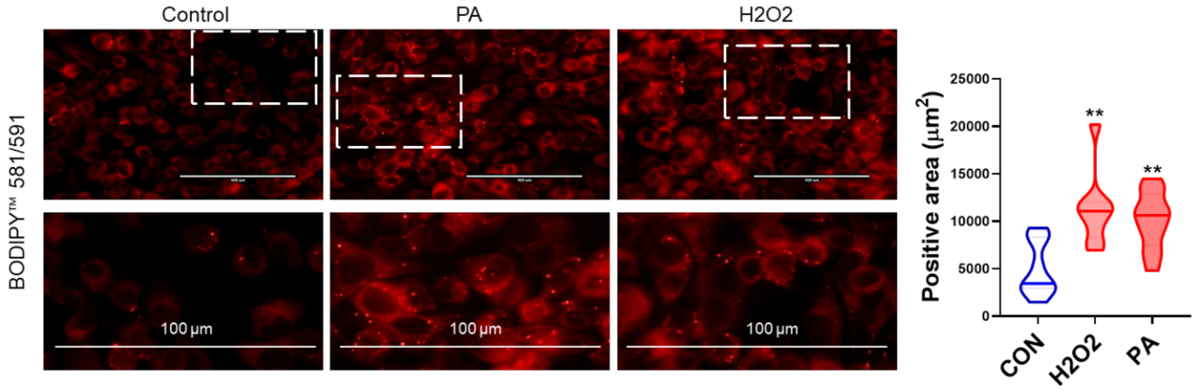

Supplementary Figure 1. Lipid ROS accumulation in HepG2 cells treated with PA, DCA, and H_2O_2 and lipid peroxidation was confirmed using BODIPY™ 581/591. Original magnification, 100 x, Scale bar = 200 μm. Data is expressed as the mean ± SE of three sets. P values were determined using one-way ANOVA post hoc Bonferroni test (** $P < 0.01$, $n = 3$).

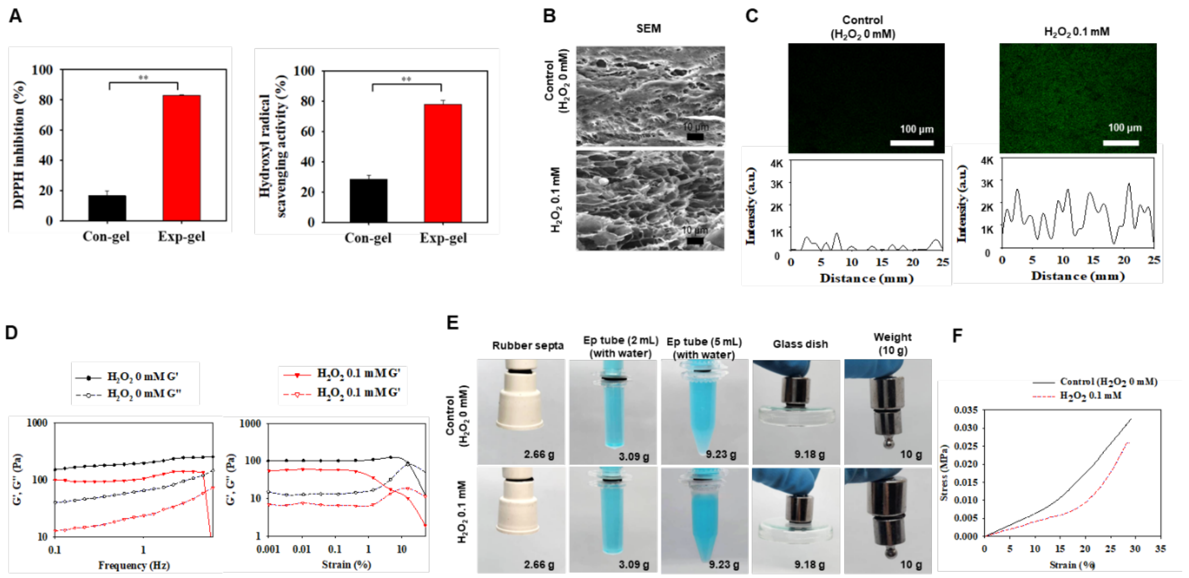

Supplementary Figure 2. (A) Antioxidant activity was evaluated by performing DPPH and hydroxyl radical scavenging assays on Con-gel and Exp-gel formulations (n = 5). ** P < 0.01. **(B)** SEM images of Exp-gel under treatment condition of H_2O_2 0 mM and H_2O_2 0.1 mM. **(C)** ROS-sensitive fluorescence recovery in Exp-gel under H_2O_2 treatment. Intensity profiles and confocal images confirm fluorescence restoration due to MnO_2 degradation in Exp-gel. Scale bar = 100 µm. **(D)** Frequency sweep (0.1-5 Hz), and (B) Streain sweep (0.001-50 %) of Exp-gel before and after H_2O_2 treatment. **(E)** Adhesion strength of Exp-gel on various substrates with and without H_2O_2 treatment. Exp-gel adheres to rubber, plastic, glass, and metal, supporting up to 10 g with maintained adhesion after ROS exposure. **(F)** Compression strength (0-30 % strain) of Exp-gel before and after H_2O_2 treatment.

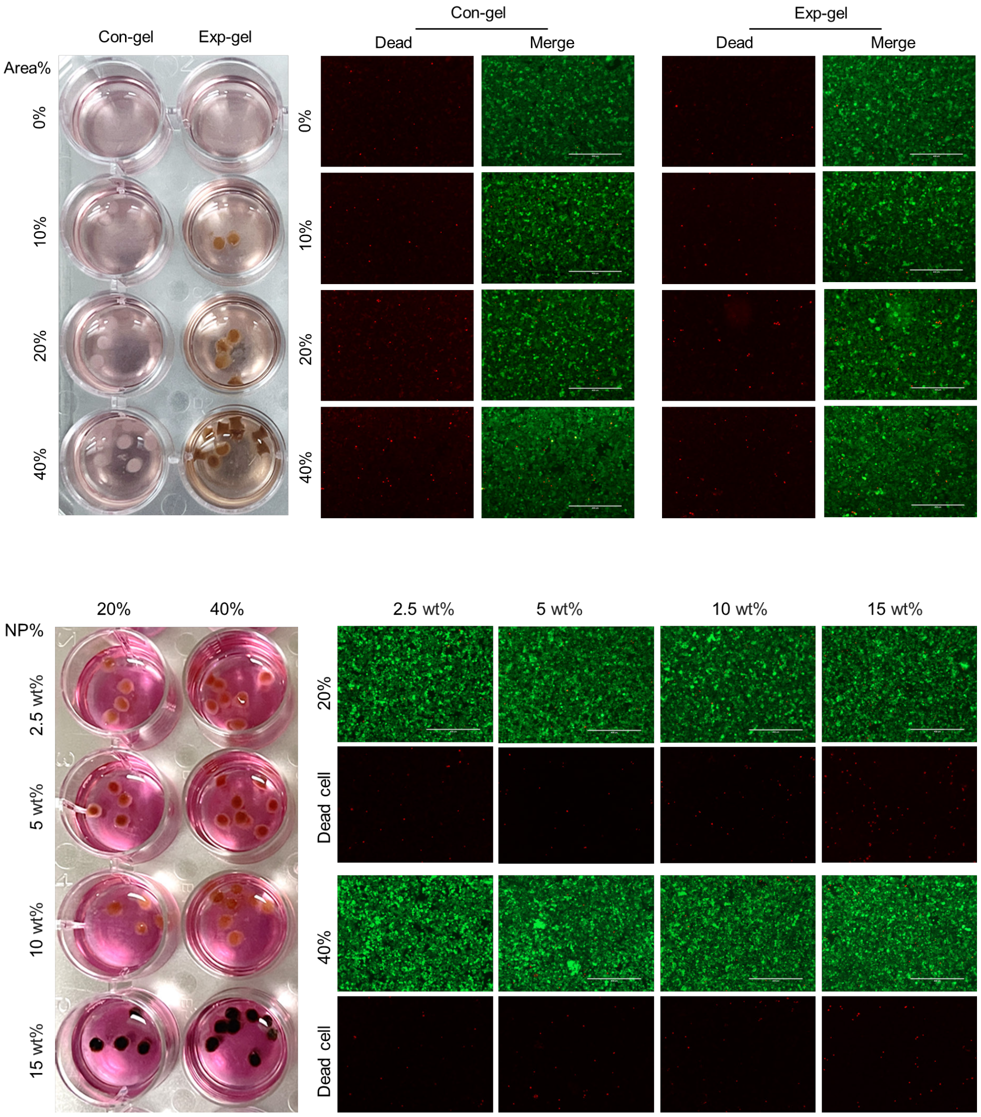

Supplementary Figure 3. HepG2 cells were cultured with hydrogels at various doses and NP percentages. Live/dead staining was then performed to assess cell viability. Images were captured at 200 x magnification (scale bar = 200 μm).

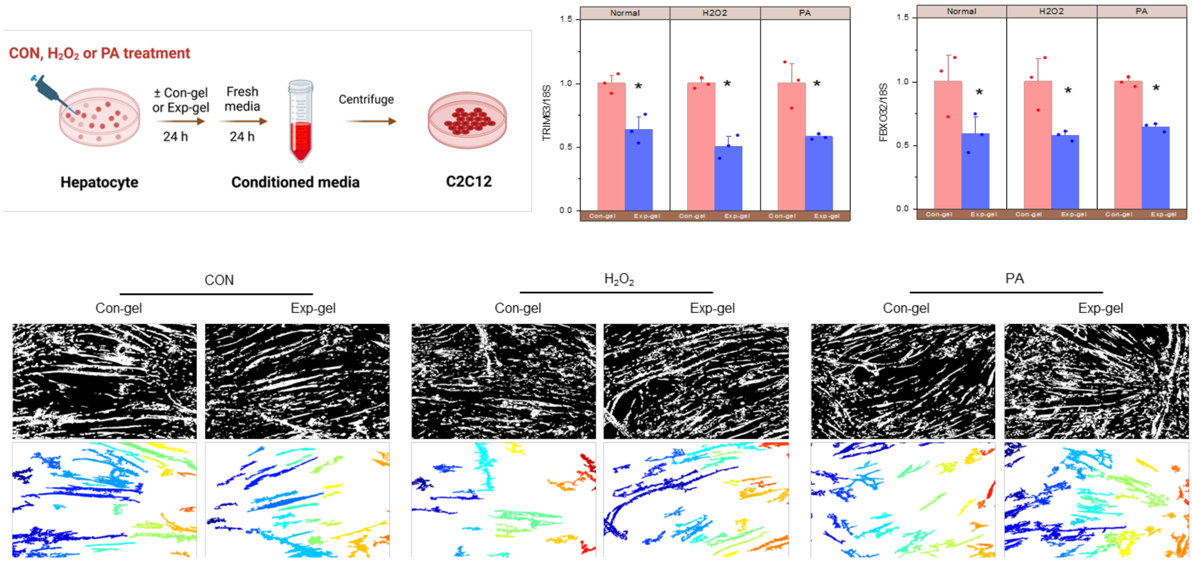

Supplementary Figure 4. Effect of conditioned media from H_2O_2 and PA-treated HepG2 on C2C12 cells. Myotube size and atrophy marker expression are compared between Exp-gel and Con-gel treatments, with Exp-gel demonstrating protective effects. Original magnification, 200 x. Data is expressed as the mean ± SE. P values were determined using unpaired two-tailed Student′s t-test. n = 3, * $P < 0.05$. Source data are provided as a Source Data file.

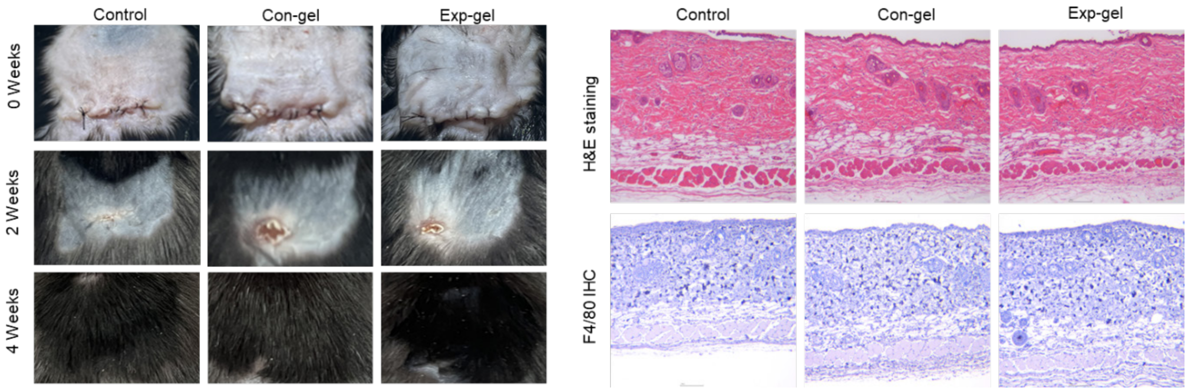

Supplementary Figure 5. Long-term in vivo biocompatibility and immune response of Exp-gel. Exp-gel was subcutaneously implanted in mice and evaluated after 4 weeks. Representative H&E staining of skin tissue at the implant site demonstrates minimal inflammatory cell infiltration. F4/80 immunohistochemistry was performed to assess macrophage infiltration, revealing a limited immune response. Scale bar = 100 μm.

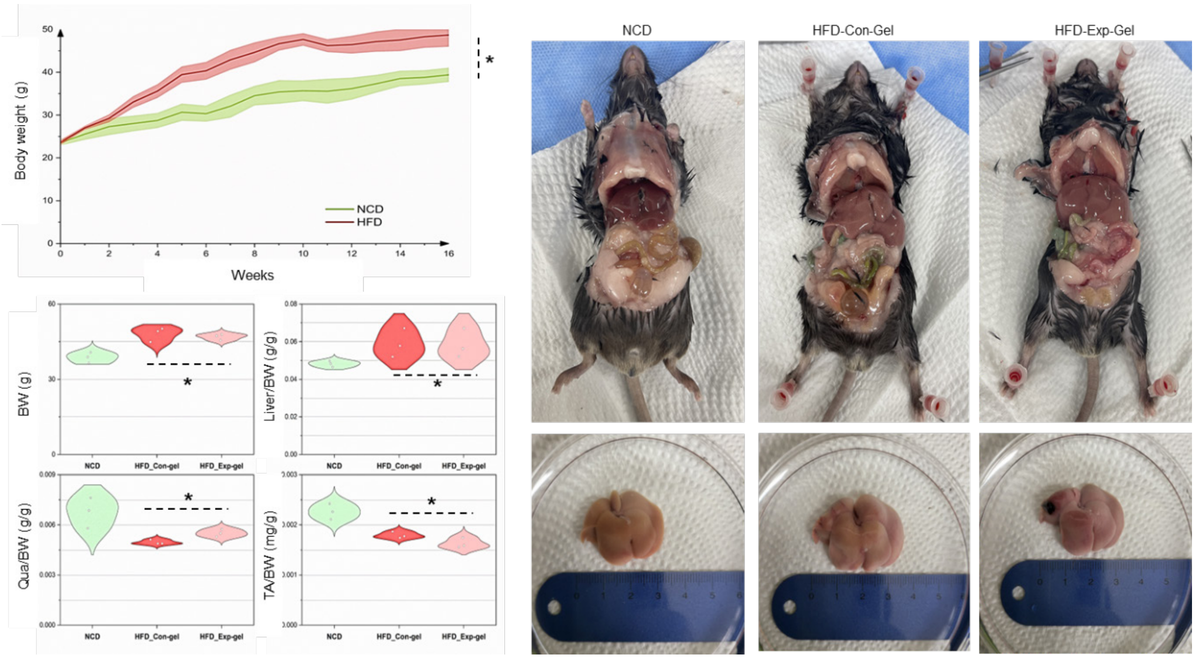

Supplementary Figure 6. Body weight tracking of mice under NCD and HFD. Violin plots of body weight, liver weight, and muscle weight ratios in NCD and HFD mice treated with Con-gel or Exp-gel, n = 3. Representative mice in each treatment group, depicting gross images of whole-body shape (top) and liver tissue (bottom), n = 3. Data is expressed as the mean ± SE. P values were determined using one-way ANOVA post hoc Bonferroni test: * $P < 0.05$.

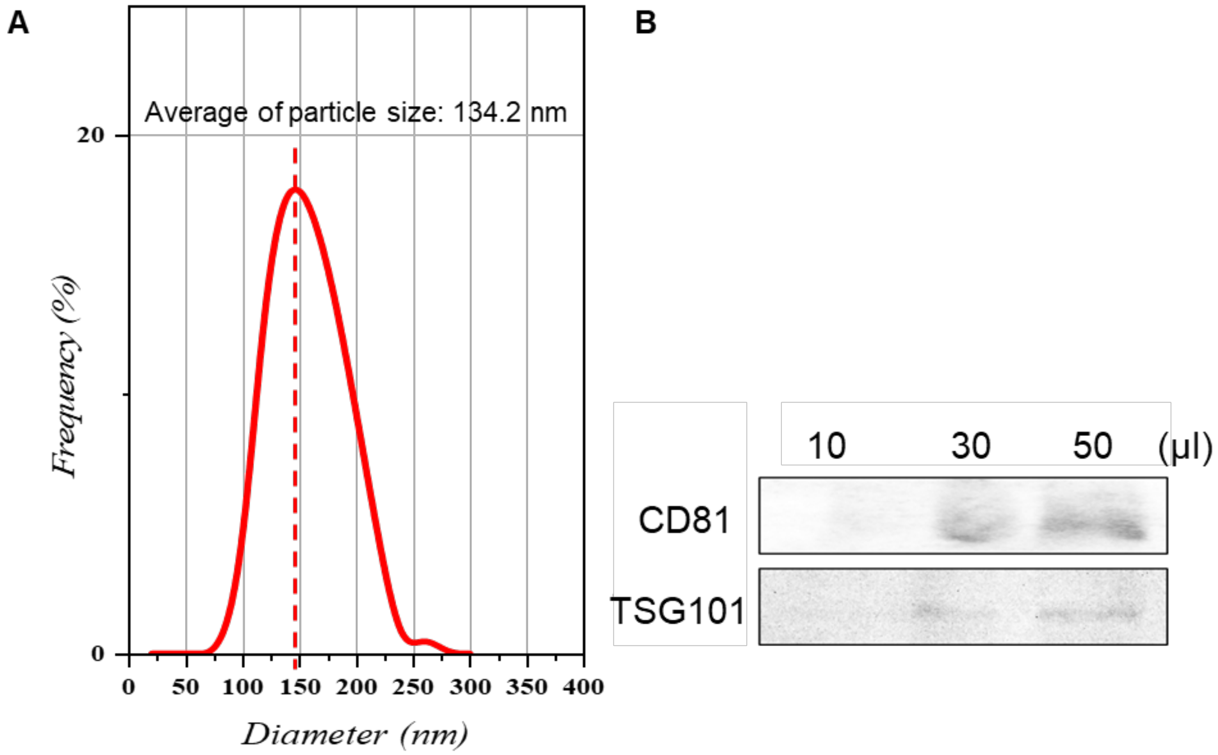

Supplementary Figure 7. Characterization of isolated exosomes. (A) Dynamic light scattering (DLS) analysis. (B) Western blot analysis of CD81 and TSG101.

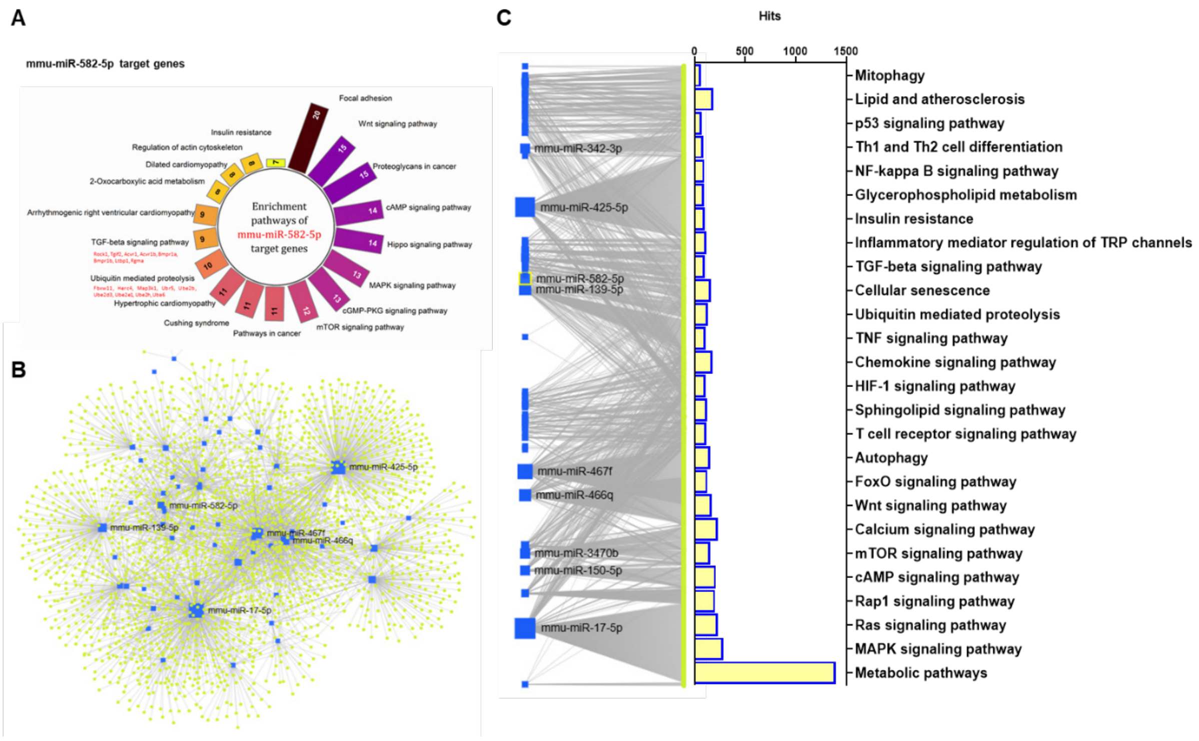

Supplementary Figure 8. Pathway enrichment and network analysis of miR-582-5p target genes.

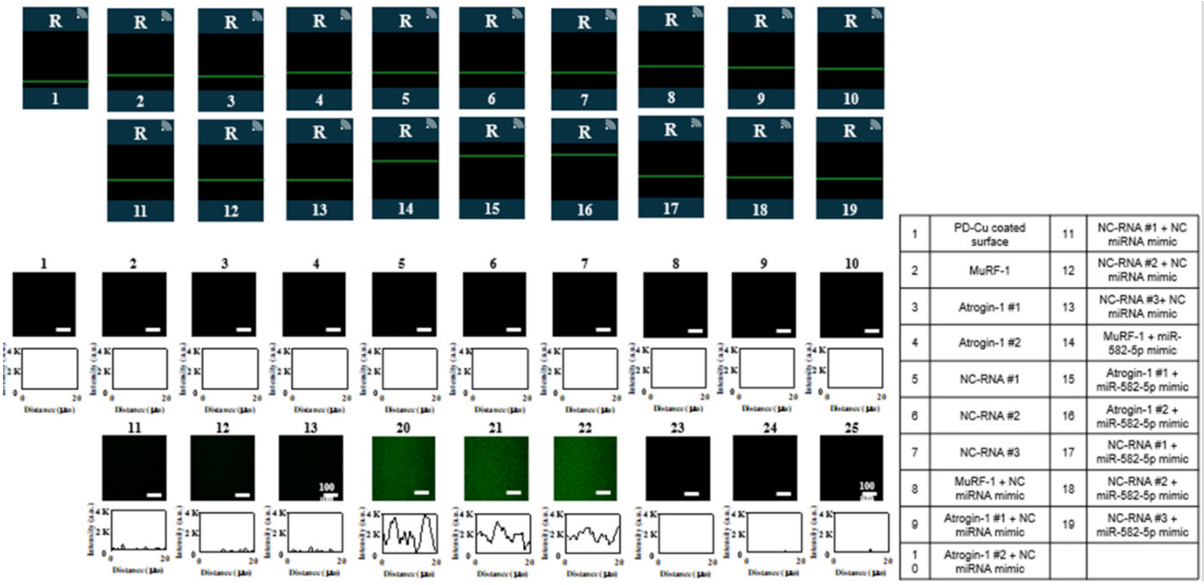

1	PD-Cu coated surface	11	NC-RNA #1 + NC miRNA mimic
2	MuRF-1	12	NC-RNA #2 + NC miRNA mimic
3	Atrogin-1 #1	13	NC-RNA #3+ NC miRNA mimic
4	Atrogin-1 #2	14	MuRF-1 + miR-582-5p mimic
5	NC-RNA #1	15	Atrogin-1 #1 + miR-582-5p mimic
6	NC-RNA #2	16	Atrogin-1 #2 + miR-582-5p mimic
7	NC-RNA #3	17	NC-RNA #1 + miR-582-5p mimic
8	MuRF-1 + NC miRNA mimic	18	NC-RNA #2 + miR-582-5p mimic
9	Atrogin-1 #1 + NC miRNA mimic	19	NC-RNA #3 + miR-582-5p mimic
10	Atrogin-1 #2 + NC miRNA mimic		

Supplementary Figure 9. Electrochemical impedance spectroscopy (EIS) confirming miR-582-5p's interaction with atrophy-related targets and off-targets**.**
